# An Electrostatic Funnel in the GABA-Binding Pathway

**DOI:** 10.1371/journal.pcbi.1004831

**Published:** 2016-04-27

**Authors:** Timothy S. Carpenter, Felice C. Lightstone

**Affiliations:** Biosciences and Biotechnology Division, Physical and Life Sciences Directorate, Lawrence Livermore National Laboratory, Livermore, California, United States of America; Institut Pasteur, FRANCE

## Abstract

The γ-aminobutyric acid type A receptor (GABA_A_-R) is a major inhibitory neuroreceptor that is activated by the binding of GABA. The structure of the GABA_A_-R is well characterized, and many of the binding site residues have been identified. However, most of these residues are obscured behind the C-loop that acts as a cover to the binding site. Thus, the mechanism by which the GABA molecule recognizes the binding site, and the pathway it takes to enter the binding site are both unclear. Through the completion and detailed analysis of 100 short, unbiased, independent molecular dynamics simulations, we have investigated this phenomenon of GABA entering the binding site. In each system, GABA was placed quasi-randomly near the binding site of a GABA_A_-R homology model, and atomistic simulations were carried out to observe the behavior of the GABA molecules. GABA fully entered the binding site in 19 of the 100 simulations. The pathway taken by these molecules was consistent and non-random; the GABA molecules approach the binding site from below, before passing up behind the C-loop and into the binding site. This binding pathway is driven by long-range electrostatic interactions, whereby the electrostatic field acts as a ‘funnel’ that sweeps the GABA molecules towards the binding site, at which point more specific atomic interactions take over. These findings define a nuanced mechanism whereby the GABA_A_-R uses the general zwitterionic features of the GABA molecule to identify a potential ligand some 2 nm away from the binding site.

## Author Summary

Neurotransmitters convey signals from one neuron to the next and are indispensable to the functioning of the nervous system. These small molecules bind to receptors to exert their action. One of the most important neurotransmitters is γ-aminobutyric acid (GABA), which binds to its type A receptor to exert an inhibitory influence on the neuron. Many drugs, both medicinal and nefarious, bind to these neuroreceptors and alter the balance of neuronal signals in the brain. There is a fine balance between these drugs eliciting the desired effect, and causing unwanted and sometimes irreversible alterations in neural behavior. To study this critical binding event, we are using computational simulations to observe precisely how the GABA molecule binds to its type A receptor (GABA_A_-receptor). One hundred individual simulations were carried out where GABA was placed near the binding site and then allowed to freely bind to the GABA_A_-receptor. Binding occurred in 19 of these simulations. Statistical analysis of these binding simulations reveals the consistent pathway taken by GABA molecules to enter the binding site. This improved understanding of the binding event enables development of safer medicinal neuroactive drugs and countermeasures for effects of neuronal chemical trauma.

## Introduction

The neurotransmitter γ-aminobutyric acid (GABA) is the brain’s major inhibitory neurotransmitter, which binds to the GABA type A receptors (GABA_A_-Rs). These GABA_A_-Rs are ‘Cys-loop receptors’ in the pentameric ligand-gated ion channel (pLGIC) superfamily. Cys-loop receptors are so named due to a well-conserved 13-residue loop that is formed between two cysteine (Cys) residues that are connected via a disulfide bond. Upon agonist (GABA) binding, the channel of the GABA_A_-R opens and increases the intraneuronal chloride ion concentration, hyperpolarizing the cell and inhibiting transmission of the nerve action potential.

GABA_A_-Rs are heteropentamers that are composed of many different combinations of distinct subunit gene products (α_1–6_, β_1–3_, γ_1–3_, δ, ε, π, and ρ_1–3_). While the most common GABA_A_-R subtype in the brain is the α_1_β_2_γ_2_ combination (comprising two α_1_-subunits, two β_2_-subunits, and a single γ_2_-subunit) [1, 2], this study will focus entirely on a minor subclass of GABA_A_-Rs that contain a δ-subunit instead of a γ-subunit. The δ-containing GABA_A_-Rs comprise only 5–10% of the total GABA_A_-Rs in the brain [3]. They are mostly located *away* from the synapses [4, 5] and are thought to be involved in the constantly active ‘tonic’ GABAergic current [6, 7]. While only comprising a fraction of the total GABA_A_-Rs in the brain, the α_6_β_3_δ receptor is one of the most highly GABA (and ethanol) sensitive receptors [8], making it the ideal GABA_A_-R subtype for studying ligand binding.

Existing structural and biochemical data show that the GABA_A_-R subunits combine to form an ion channel through the membrane via a pore down the center of the pentamer. Currently, no experimental structure of a heteropentameric GABA_A_-R is available. A structure for a homomeric GABA β_3_ pentamer has been recently released [9], but despite being the first (and so far, only) high-resolution structure resolved, it is a non-physiologically occurring construct. This β_3_ pentamer possesses the same structural architecture as described in previous extensive comparison studies [10, 11]. Each monomer is comprised of three domains; the extracellular ligand-binding domain (LBD) is comprised of a ‘β-sandwich’ structure; the transmembrane (TM) domain is composed of four helices; and a cytoplasmic domain of relatively unknown structure forms between TM helices 3 and 4. The LBD of each subunit consists of a ‘principal’ (+) and ‘complementary’ (–) side. The GABA binding site is formed by a cleft between the α– and β+ subunit interfaces [12, 13]. The specific GABA binding site within this cleft has been well defined, with explicit key residues identified in experimental literature (α_6_F65, α_6_R132, β_3_L99, β_3_E155, β_3_R196, β_3_Y205, and β_3_R207) [14–20]. We have previously detected these residues as critical to GABA binding in our own computational studies [21]. While most of the α– and β+ subunit interfaces form the ‘sides’ of the binding pocket, the β-subunit C-loop folds over the top of the pocket to act as a ‘cap’ or ‘roof’ to the binding site. The movement of this C-loop has been linked to the activation mechanism of Cys-Loop receptors [22], and a closed C-loop has long been thought necessary for obtaining the active state of Cys-Loop receptors [23–25]. Throughout the rest of the paper we will refer to the region of the LBD that is closer to the TM helices than the C-loop as being ‘below’ the binding site, and the region of the LBD that is further from the TM helices than the C-loop as being ‘above’ the binding site.

A recent gating model in pLGICs suggests that the activation proceeds via a “conformational wave” that starts in the ligand-binding site (notably loops A, B, and C), and then propagates to the LBD/TM interface (the β_1_-β_2_ loop and the Cys-Loop) and finally moves to the TM helices (firstly the M2 helix) to cause the ion pore to open [26, 27]. This activation model was validated further with coarse-grained normal-mode analyses of the ELIC and GLIC structures to calculate a closed to open state transition pathway [28]. Conversely, one of the latest computational studies [29] observed a pLGIC open to closed transition pathway upon agonist unbinding. The agonist unbinding was mediated by opening of the C-loop and caused a significant reorientation of the β-sandwiches in the LBD that tilted outward. This rearrangement lead to the β_1_–β_2_ loop repositioning at the LBD/TM domain interface, resulting in an inward displacement of the M2–M3 loop and an inward tilting of the pore-lining helices to shut the ion pore. This study also suggests that C-loop opening is necessary but not sufficient for agonist unbinding [29].

Consequently, details about the agonist binding site are known, as well as how agonist binding triggers the activation mechanism that leads to channel opening. However, the vast majority of the critical residues that line the binding pocket are obscured by the C-loop in the available structures, leaving very little of the ‘binding site’ exposed. Thus, questions still remain as to how GABA recognizes this binding site, and what is the pathway by which GABA gets into the binding site.

In order to investigate these open questions, we attempt to map the binding pathway of GABA using unbiased molecular dynamics (MD) simulations. Using extensive MD simulations, GABA molecules are isolated and a consensus pathway to binding the receptor is determined. Fundamental driving forces that control the binding pathway are identified and analyzed, revealing the need to expand our scope for what we consider as important residues and regions for ligand-binding.

## Results

### Categorization of simulations

The initial criteria used for defining the ‘binding state’ of GABA in the simulations is the distance between the center of mass (COM) of the GABA molecule and the COM of those residues that line the binding pocket (α_6_F65, α_6_R132, β_3_L99, β_3_E155, β_3_R196, β_3_Y205, and β_3_R20). In order to spatially define when a GABA molecule is within the binding pocket, we ran analysis on a control simulation, where GABA is directly docked into the binding site. This 20 ns simulation shows that the GABA molecule equilibrated to a consistent position within the pocket, with its COM ~0.62 ± 0.06 nm from the COM of the binding site. Thus, we define a GABA molecule that has a COM <0.70 nm from the COM of the binding site as being *in* the pocket. Given that the GABA molecule itself is ~0.65 nm long, when the GABA COM is within 1.3 nm of the binding site COM, then at least *part* of the GABA molecule is within the region of the binding pocket. When the GABA COM is within 1.0 nm of the binding site COM, then the majority of the GABA molecule is within the binding pocket region.

**Fig 1:**
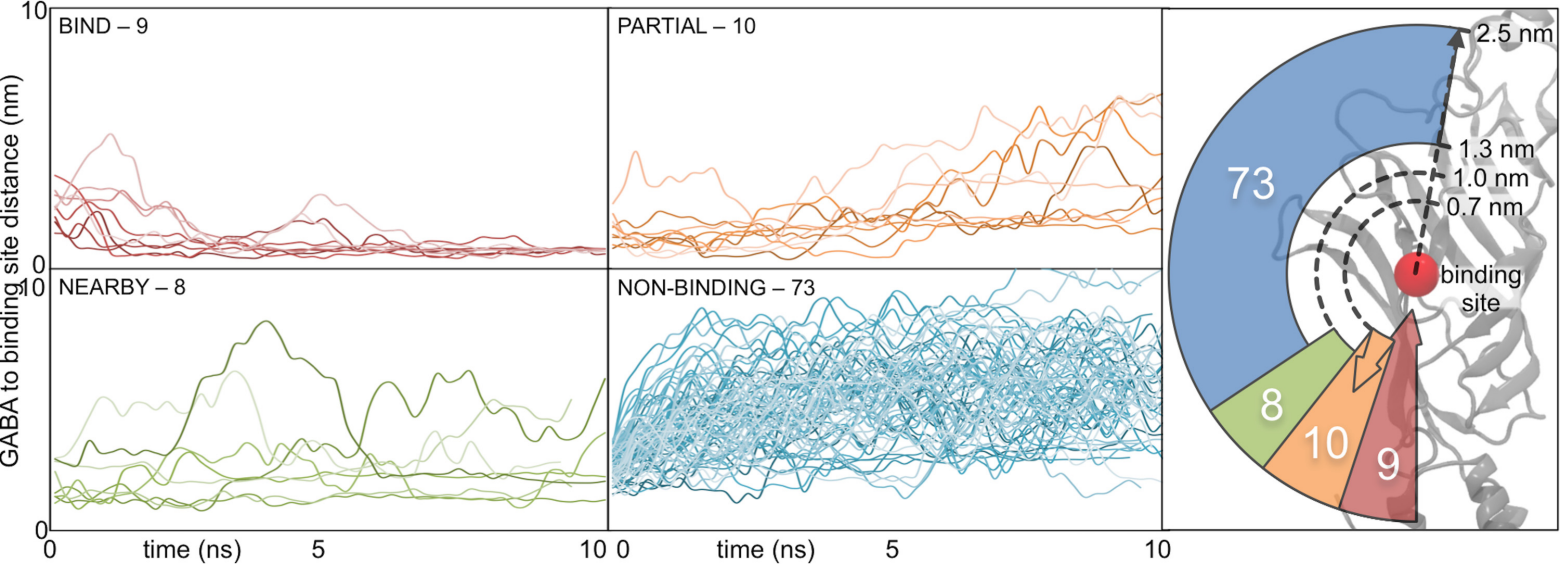
Breakdown of GABA simulations by category. The 100 independent GABA simulations are broken down into four categories based upon their distance from the GABA binding site. Those that enter the binding site (< 0.70 nm from the binding site COM) and remain there fall into the ‘BIND’ category (red). Those that enter the binding site and leave again fall into the ‘PARTIAL’ category (orange). Those that partially enter the binding site (< 1.3 nm from the binding site COM) fall into the ‘NEARBY’ category (green). Those that do not even partly enter the binding site (never < 1.3 nm from the binding site COM) fall into the ‘NON-BINDING’ category (blue). The radial representation on the right shows the populations of each of the categories (with the corresponding color) and the relative proximity they reach to the binding site COM (shown as a red circle). The number next to the category is the number of simulations that are in that category.

Using this metric for the ‘binding state’ and cut-off values to indicate binding in the pocket, the simulations fall into four distinct groups (**Fig 1**). The largest group of simulations is when GABA is ‘**NON-BINDING**’ (73 simulations)–defined as those that never get closer than 1.3 nm to the binding site COM. Eight of the simulations are when GABA binds ‘**NEARBY**’–defined as GABA getting partially within the binding site (< 1.3 nm) without fully reaching within the binding site (> 0.70 nm). Finally, nineteen simulations that show GABA ‘binding’–defined as the GABA COM getting closer than 0.70 nm from the binding site COM–are subdivided into two categories: 1) ‘**BIND**’ (nine simulations) where GABA remains within the binding site, and 2) ‘**PARTIAL**’ (10 simulations) where GABA reaches the 0.70 nm cut-off, but then moves away again. Through the remainder of this article, we refer to the simulations as ‘**NON-BINDING**’, ‘**NEARBY**’, ‘**PARTIAL**’, and ‘**BIND**’. All simulations in ‘**NEARBY**’, ‘**PARTIAL**’, and ‘**BIND**’ are used in subsequent analysis (as described in the **Methods**). A random subset of 20 of the 73 simulations is chosen as a ‘**NON-BINDING**’ sample to analyze the non-binders.

**Fig 2:**
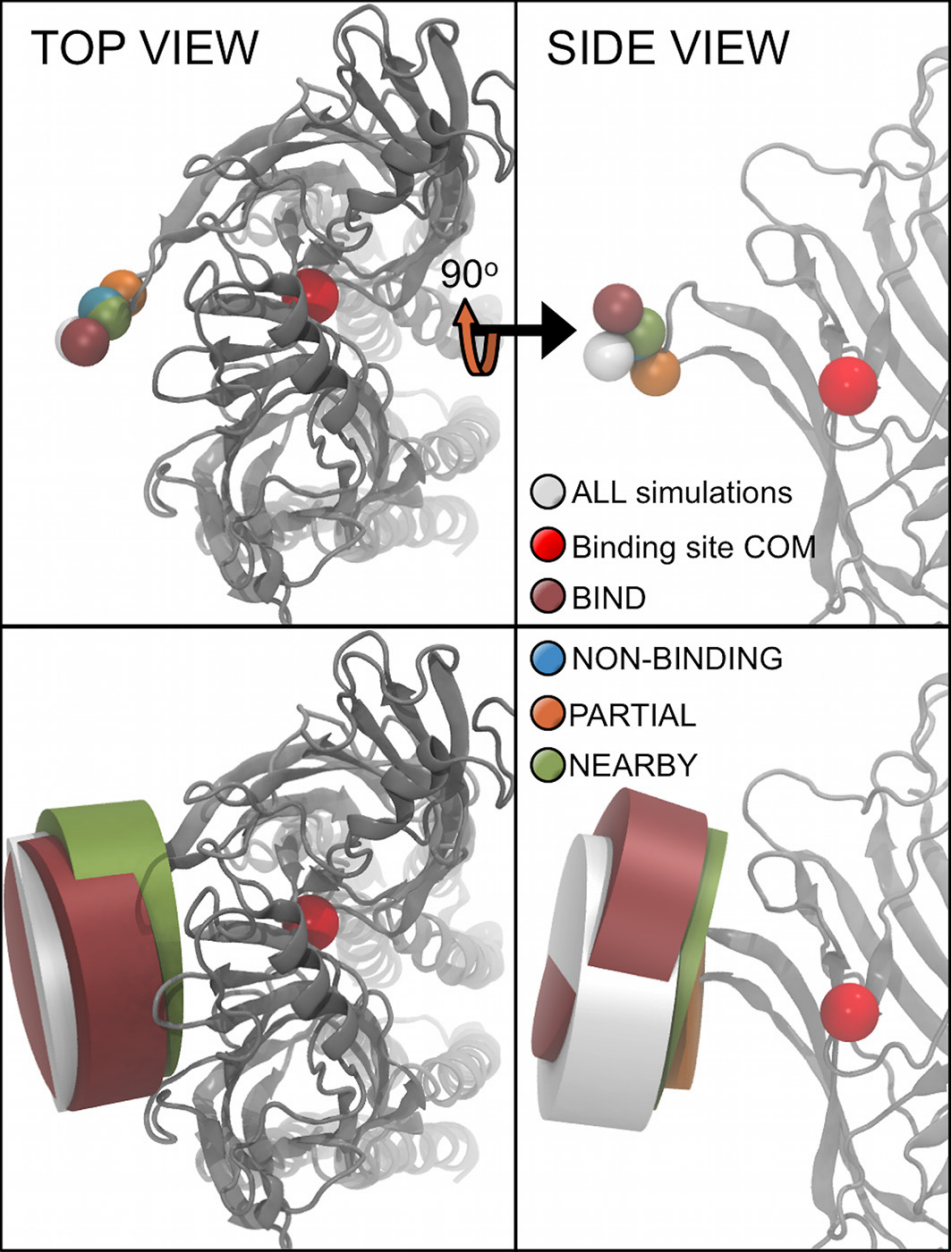
Average starting positions of the GABA molecules in the different simulation categories. The average starting positions of the GABA molecules in each simulation category are shown as spheres (top row). The GABA binding site COM is also indicated. The distribution of molecules within one standard deviation from the average is also shown (bottom row).

It is important that the starting positions of the GABA molecules do not influence their binding, and confirmation of independent starting positions is vital. Thus, three methods were used to verify that the initial starting positions of the GABA molecules in our simulations did not bias the outcome of the results. Firstly, the average starting positions of all the GABA molecules within each category were calculated and are represented relative to the protein and the binding site COM (**Fig 2**). Not only do the average starting positions all occupy a similar location, but the standard deviations of the GABA position in each category are similar in size and completely overlap one another. This indicates that the quasi-random initial starting positions of the GABA molecules had no bias on the molecule’s probability to enter the binding site. Secondly, the average initial positions are all approximately level with the ‘height’ (z-coordinate position) of the binding site COM, as well as closely aligned with the vector from the binding site COM directly away from the protein; indicating no bias in any particular *direction* relative to the binding site. Thirdly, the initial movement of the GABA molecules was measured as a movement towards or away from the protein (**S1 Fig**). This movement shows no bias towards the protein and displays random behavior. All three methods confirm that the initial position of the GABA molecules in each simulation do not bias the results.

### Average binding pathway

**Fig 3:**
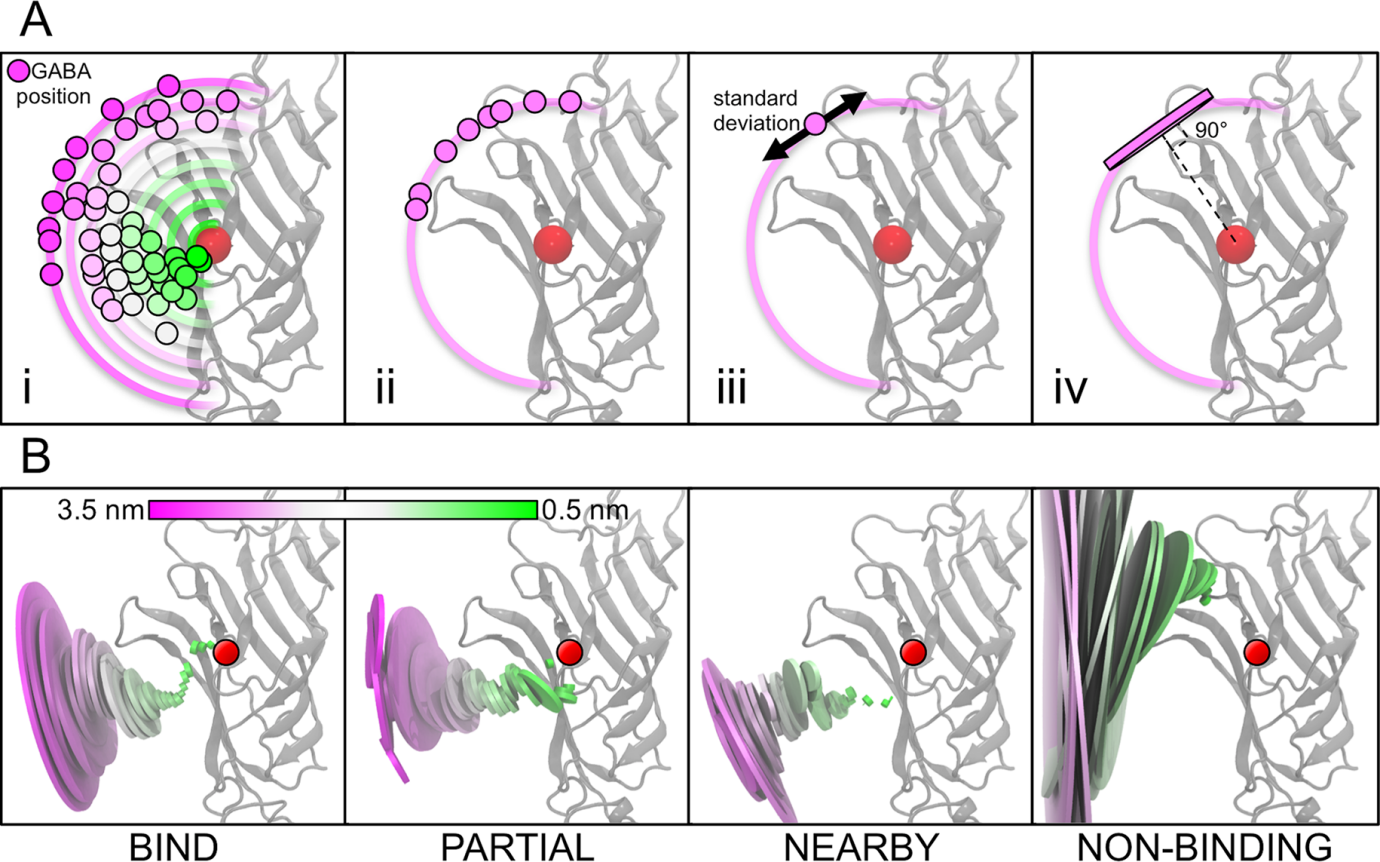
Calculation of the GABA pathway. (A) In order to determine the average location of the GABA molecules for each category, the positions of GABA are binned into windows based upon 0.1 nm increments of the GABA COM to binding site COM distance (i). The positions of all the GABA molecules within a particular bin (ii) are used to calculate an average position and standard deviation of GABA at that particular distance from the binding site COM (iii). These data are represented graphically as a disc with a radius that is proportional to the standard deviation of the GABA positions within that bin, and with its axis aligned to the vector between the average GABA position and the binding site COM (iv). (B) The average binding pathways calculated for each of the simulation categories (L-R: BIND, PARTIAL, NEARBY, NON-BINDING) are shown. The radius of the disc is proportional to the standard deviation of GABA molecule positions at that distance from the binding site. The ligand-binding domain of the β_3_-subunit is also illustrated in a grey cartoon format. A red circle represents the binding site COM. The discs used for these figures were constructed by using the draw feature of VMD to create cylinders with defined centers along the vector between the ligand binding site COM and the ligand position COM. The radius of the cylinder is defined as the measured standard deviation at that position.

To determine the distinct pathway for GABA to bind to the GABA binding site, average positions of GABA relative to the protein within each simulation category are calculated (as described in **Fig 3A**). These average GABA positions indicate that all of the ‘binding’ (BIND, PARTIAL, NEARBY) simulations follow a similar pathway (**Fig 3B**) with comparable characteristics: 1) GABA approaches the binding site from the membrane side of the C-loop (*below*) before reaching the protein and then moves (‘flips up’) up into the binding site, and 2) the distribution of the GABA positions within the pathway is very narrow. By contrast, the NON-BINDING simulations appear to have GABA positioned further from the membrane, ‘*above*’ the binding site and have a much wider distribution of GABA positions with larger standard deviations.

**Fig 4:**
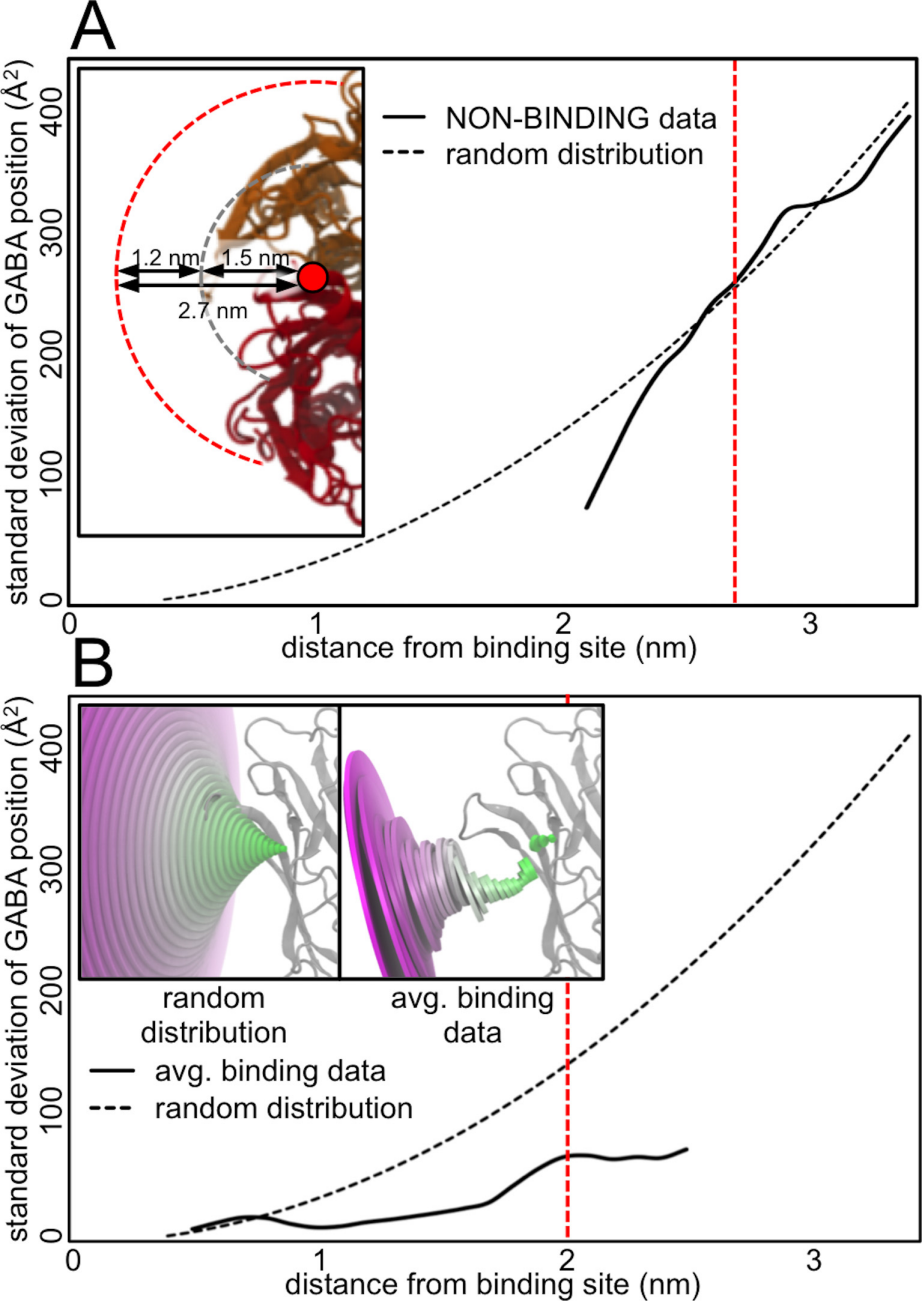
GABA position distribution compared to random distribution. (A) The standard deviation of the GABA positions from the NON-BINDING simulations is compared against the standard deviation of a hypothetical random distribution. The dashed red line indicates the position ~2.7–2.8 nm from the binding site where the GABA distribution begins to deviate from random behavior. The edge of the protein is ~1.5 nm from the binding site COM. The non-bonded van der Waals interaction cutoff used for the simulation is 1.2 nm. Thus when GABA is ~2.7 nm from the binding site, it only ‘feels’ the presence of the protein via weak long-range electrostatic effect (A–inset). (B) The average standard deviation of all the binding simulations (BIND, PARTIAL, and NEARBY) is also compared against standard deviation of a hypothetical random distribution. A visual representation of these distributions is also shown (B–inset). The dashed red line indicates where the ‘focusing’ or ‘funneling’ of the GABA distribution occurs at ~1.9–2.0 nm from binding site.

**Fig 5:**
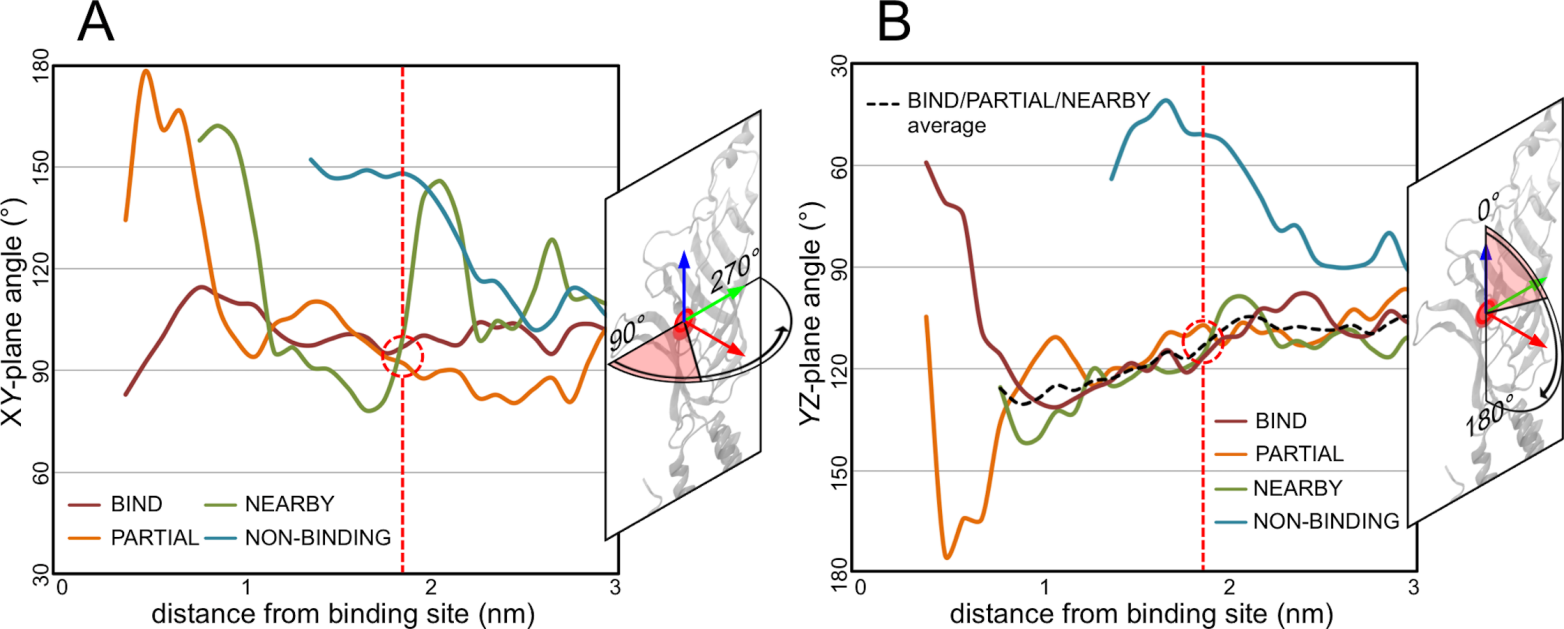
The angular positions of the GABA molecules. The average angular positions of all the GABA molecules within a simulation category are shown as a function of their distance from the binding site COM. These angles are presented for both the XY (A) and YZ (B) planes. The red dashed line indicates the distance at which the GABA molecules converge at the same point.

Although a consistent pathway for GABA molecules to approach the binding site from the membrane side of the C-loop was identified, further analysis shows this pathway is determined by influences of the protein. A theoretical non-biased “random” distribution of GABA locations was artificially generated and used to calculate the hypothetical positional standard deviations expected if GABA molecules were to approach the binding site in a completely random manner. This standard deviation data calculated from an artificially generated random distribution was used as a metric for random/non-biased GABA dispersal. The NON-BINDING simulations do indeed have GABA distributions that are comparable to these hypothetical ‘random’ distributions (**Fig 4A**). The GABA molecules in these NON-BINDING simulations begin to adopt this quasi-random and mostly-unbiased distribution behavior once they reach a distance of 2.7 nm from the binding site COM (**red line, Fig 4A**). At this distance, the GABA molecules are beyond the forcefield cut-off distance to be influenced by van der Waals interactions with the protein. As such, these GABA molecules are essentially experiencing bulk solution-like conditions with only weak, long-range electrostatic effects from the protein and thus are more randomly distributed. When the hypothetical ‘random’ distribution is compared to the average distributions seen in all the binding (BIND, PARTIAL, and NEARBY) simulations, the pathway by which GABA molecules approach the binding site is indeed far from random, as shown in **Fig 4B**. The presence of the protein greatly influences the GABA molecules to become more concentrated at specific positions as they approach the binding site. In particular, the GABA molecule positions are definitively ‘focused’ or ‘funneled’ once they reach a distance of ~1.9–2.0 nm from the binding site COM (**red line, Fig 4B**). Furthermore, deconstructing the average GABA positions into the angles they make with both the XY-plane and the YZ-plane shows that for all three of the binding categories (BIND, PARTIAL, and NEARBY), both the XY-plane angle and the YZ-plane angle converge to almost the same value when the GABA molecules are at a distance of ~1.9 nm from the binding site COM (XY-plane angle = ~95°, YZ-plane angle = ~110°) (**Fig 5**). Thus, at a distance of 1.9–2.0 nm from the binding site COM, the GABA molecules of all the binding simulations converge to this same point and remain narrowly distributed as they approach the protein past this location. We have termed this position the ‘midpoint’ in the binding pathway; a crucial checkpoint through which all the binding simulations must progress. The next stage of our analysis was to determine what factors are causing the convergence and subsequent funneling of the GABA molecules at this midpoint.

### Electrostatics drive the binding pathway

**Fig 6:**
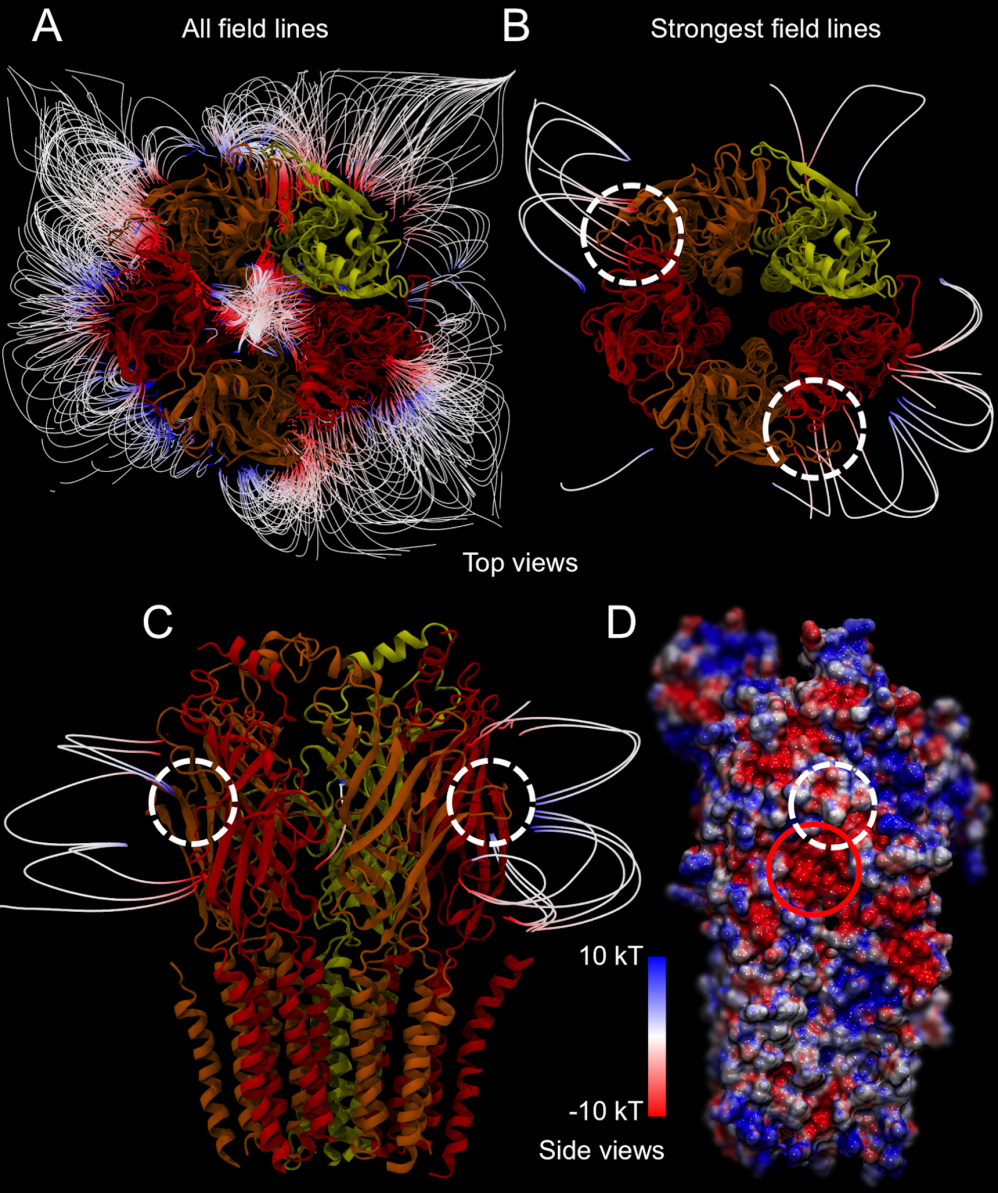
The electrostatic field surrounding the GABA_A_-R. (A) The top view of the GABA_A_-R is illustrated (α_6_-subunits in red, β_3_-subunits in orange, and the δ-subunit in yellow) with all the surrounding electrostatic field lines shown. (B) The representation of the field lines is reduced to just show the strongest field lines, which converge on the GABA binding sites (highlighted by the white dashed circles). (C) This representation is also shown from the side view. (D) Finally, the electrostatic surface of a β_3_-subunit and a α_6_-subunit is presented to illustrate the dense electronegative region just below the binding site. The binding site is highlighted by a white dashed circle, and the electronegative region is highlighted by a red circle.

Given that the GABA molecule is zwitterionic, with charged termini connected by a short hydrocarbon linker, the driving forces behind the binding pathway may be electrostatic in nature. We calculated the electrostatic potential surface of the protein to test this hypothesis. These data are also used to construct a representation of the electrostatic field that surrounds the GABA receptor (**Fig 6**). Visualization of the field lines provides an intuitive approach to identify the regional intensity and gradient of electric fields in relation to the GABA_A_-R structure. In other protein systems, such as acetylcholinesterase, the field lines around the protein are often used to interpret binding mechanisms for the positively charged acetylcholine [30, 31].

Analysis of the electrostatic field lines around the GABA receptor reveals that the strongest, most persistent areas of the field converge at two regions of the GABA receptor (**Fig 6A–6C**) that correspond to the two GABA binding sites at the α+β- subunit interfaces. Specifically, the electrostatic potential surface shows a highly electronegative region in the α+β- cleft just below the GABA binding site (**Fig 6D**), where the electrostatic field lines converge. As a comparative assessment, this same electronegative region has been observed in previously published GABA models for both the α_6_β_3_ and α_1_β_2_ clefts [21, 32, 33], as well as test models constructed using the recently published GluCl [34] and GABA β_3_ [9] homopentamer crystal structures (**S2 Fig**).

**Fig 7:**
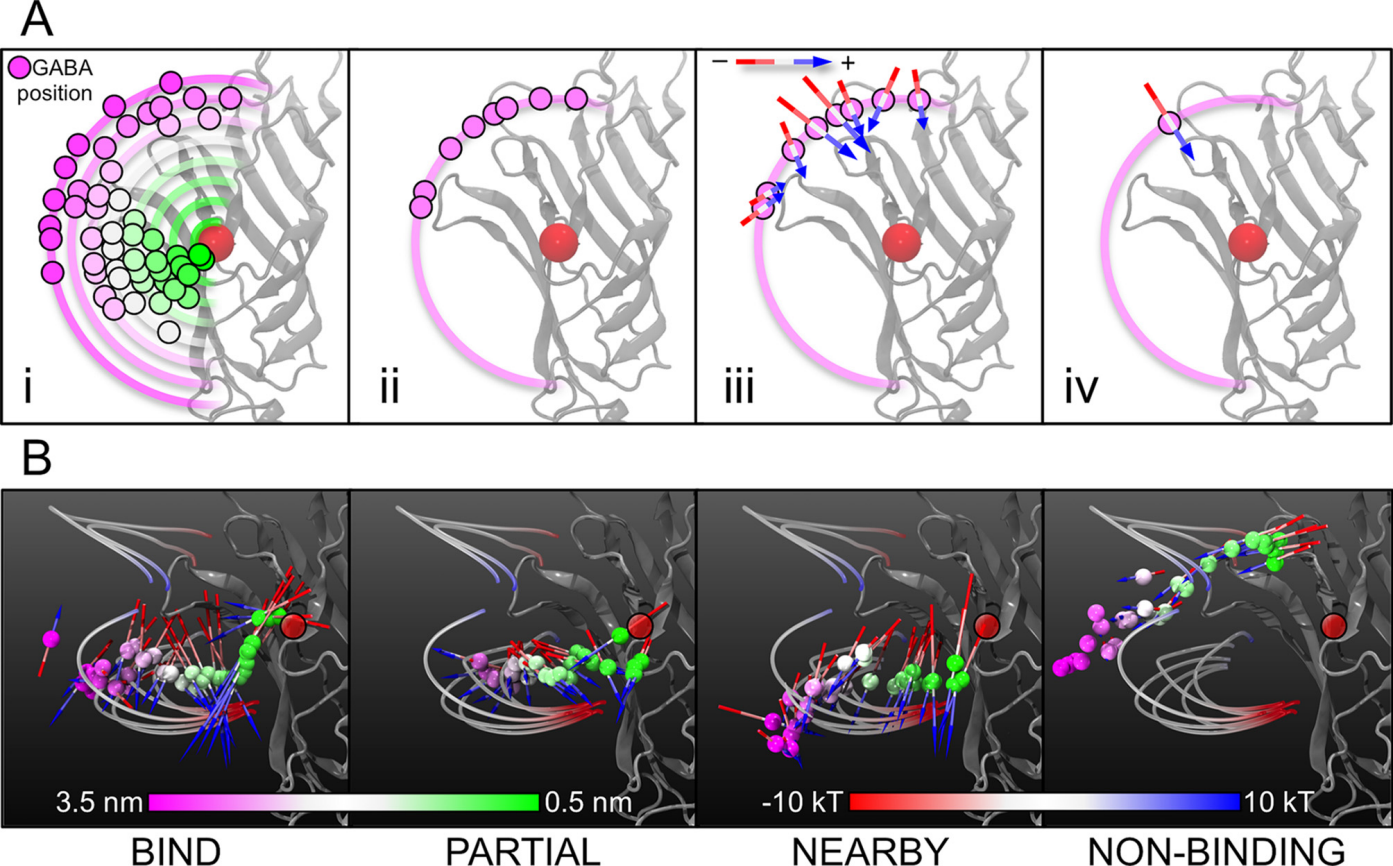
Calculation of the average dipole. (A) The influence of the electrostatic field on GABA was measured by calculating the net strength and direction of the dipole on the GABA molecules as they approach the binding site. Using the same positional bins (A.i and A.ii) as in Fig 3 (GABA molecules binned into windows based upon 0.1 nm increments of the GABA COM to binding site COM distance), the dipoles on all the GABA molecules within a particular bin were measured as vectors (A.iii) and averaged to calculate a net dipole on GABA at that particular distance from the binding site COM. These data are represented graphically as an arrow, centered on the average GABA position in that bin, pointing in the direction of the net dipole, and whose length is proportional to the dipole strength within that bin (A.iv). (B) The net dipole experienced by GABA at these positions, and their location relative to the protein and the electrostatic field is shown for each of the simulation categories (L-R: BIND, PARTIAL, NEARBY, NON-BINDING). The orientation of the dipole on the GABA molecule is represented by an arrow, with the length of the arrow proportional to the strength of the dipole. The electronegative end of the dipole arrow is colored red, while the electropositive end of the dipole is colored blue. A red circle represents the binding site COM.

The influence of the electrostatic field on GABA is measured by calculating the net strength and direction of the dipole on the GABA molecules as they approach the binding site (**Fig 7**). All of the GABA binding pathways follow the electrostatic field lines with the dipoles of the GABA molecules strongly aligned within the field. It appears that the GABA molecules are being ‘swept along’ within the electrostatic field. By stark contrast the GABA positions in the NON-BINDING simulations do not appear to correlate with the electrostatic field lines, and the net dipole strength is much reduced. Indeed, at distal positions > 2.5 nm from the GABA binding site, there is no net dipole (**‘NON-BINDING’, Fig 7B**). At these positions the GABA molecules are randomly oriented, and as such any dipole on the molecules will cancel out.

By examining the overall properties of the GABA molecules, we observe that a large change in behavior occurs at the pathway ‘midpoint’ ~1.9–2.0 nm from the binding site COM. It is at this position when the GABA molecules enter the electrostatic field. Entry into the electrostatic field causes the GABA molecules to become more converged in their *position* and their *orientation*, as they become aligned within the electrostatic field. Thus, the field causes the standard deviation of the GABA position to decrease, and the average GABA dipole strength to increase (**S3 Fig**).

### Statistical analysis of the pathway

One of the critical junctures in our pathway is the midpoint ~1.9–2.0 nm from the binding site COM, where the GABA molecules enter the electrostatic field. To emphasize the essential importance of this point in the pathway, the distance between GABA and the midpoint was measured for each of the 100 independent simulations (**S4 Fig**). GABA molecules are considered as having reached the midpoint when the distance between the GABA COM and the midpoint coordinates is <1.0 nm (as the midpoint is actually a ‘region’ with a variability of ~0.5–0.6 nm). Using this metric, 28 of the 100 simulations reach the midpoint, whereas 72 do not. Of the 28 simulations that reach the midpoint, 24 of them (86%) go on to enter the binding region. Of the 72 simulations that *do not* reach the midpoint, only 3 enter the binding region. Thus, of the 27 total simulations (BIND, PARTIAL, and NEARBY) where GABA enters the binding region, 24 (89%) proceed via the midpoint, indicating that the midpoint is indeed a crucial checkpoint that must be reached before progressing to the binding site.

**Fig 8:**
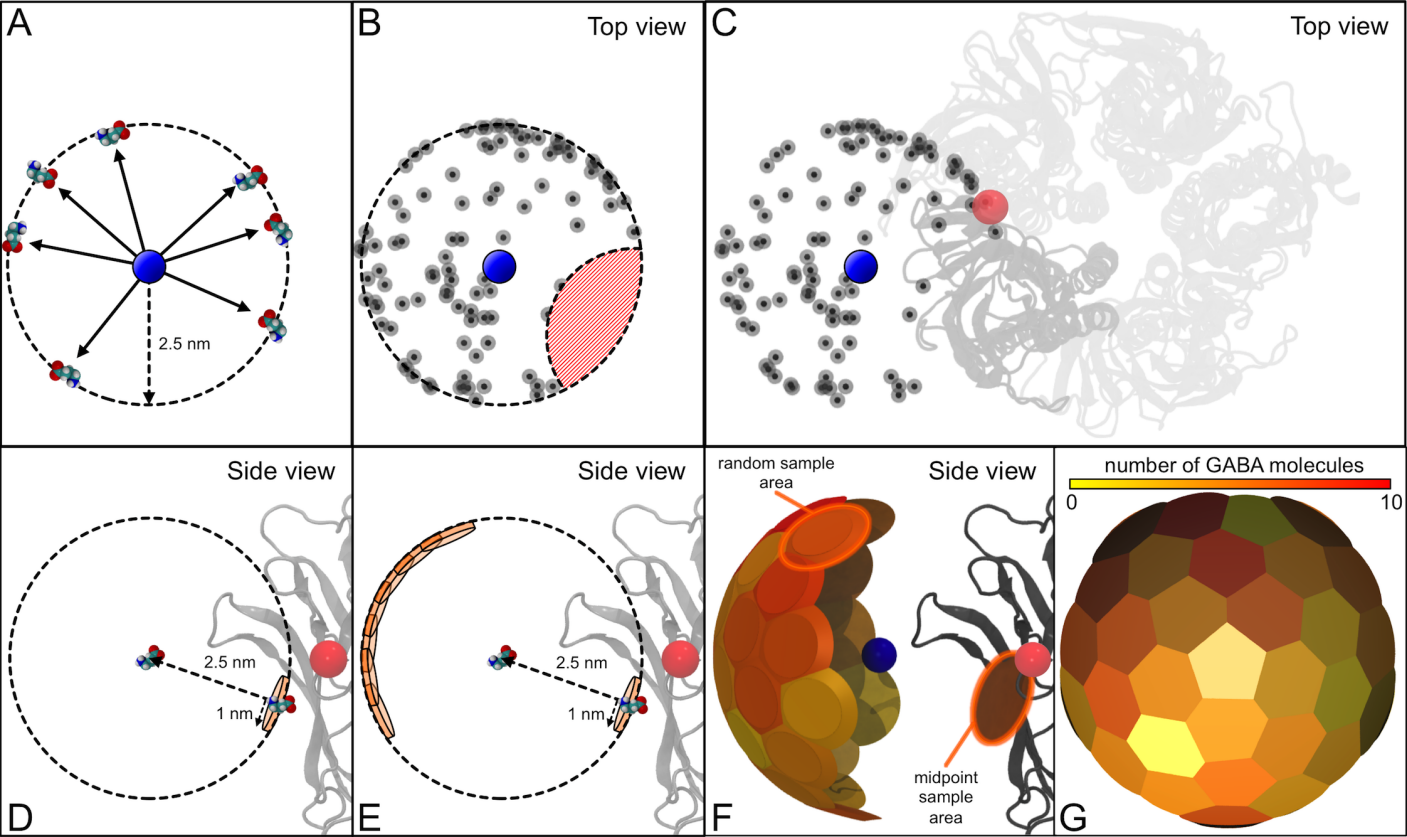
Preliminary dispersion of GABA molecules. The ‘midpoint’ is ~2.5 nm from the average starting position of the GABA molecules. To compare the initial distribution of the GABA molecules in the 100 independent simulations, their positions were calculated when they first reach a point 2.5 nm from the average starting position (A), essentially the surface of a sphere of radius 2.5 nm. The average starting position of the GABA molecules is shown as a blue circle. These locations where the GABA molecules first reach 2.5 nm from the starting position reveal that not all of the surface area of the 2.5 nm sphere is actually accessible (B). The red hashed area is devoid of GABA molecules. This is due to the region being occluded by part of the GABA_A_-R itself (C), specifically, the α_6_-subunit that comprises part of the binding site. This α_6_-subunit is shown in dark grey. The relative position of the GABA binding site COM is illustrated as a red circle, and the rest of the protein is shown in light grey, from a viewpoint looking down on the protein. Thus, excluding this protein-occluded region, the calculated accessible area on this 2.5 nm shell is ~73 nm^2^. The number of GABA molecules that first reach the 2.5 nm shell within 1.0 nm of the midpoint was calculated (D). The binding site COM is shown as a red circle, and the β_3_-subunit of the binding site is shown in grey. We are essentially measuring the number of GABA molecules within a specific circle of radius 1 nm on the surface of a sphere of radius 2.5 nm. 32 additional, ‘random’ overlapping sampling points were measured (E), and represented as colored patches on the surface of the 2.5 nm sphere (F and G). The chance of a molecule randomly reaching any sample point is ~4.3%. This is the area of sample point (approximately a circle of radius 1 nm–3.14 nm^2^) as a fraction of the available area (calculated as ~73 nm^2^). The numbers measured for the sample points (4.55 ± 1.80) portrayed in (F) and (G) indicate that there is no preference for the GABA molecules to initially travel to the midpoint, and that the positions of the GABA molecules are consistent with a random distribution.

Further analysis was carried out to look at the probability of GABA reaching the midpoint. The midpoint is ~1.9–2.0 nm from the binding site COM, but ~2.5 nm from the average GABA starting position. Thus, to test the initial dispersal of the GABA molecules, each of the simulations was monitored until the GABA molecule reached a distance of 2.5 nm from the average starting position (**Fig 8A–8C**). The coordinates at which the GABA molecules first reach this ‘2.5 nm spherical shell’ were measured and cross-referenced with those that are within 1.0 nm of the midpoint (**Fig 8D**). Of the 100 simulations, six GABA molecules first reach the 2.5 nm shell within the vicinity of the midpoint. Thirty-two equally distributed ‘random’ sample points on the 2.5 nm shell were also analyzed (**Fig 8E–8G**). These points are positioned on the opposite side of the 2.5 nm shell to the midpoint (and are away from the protein), and are ~1.0 nm from each other. The average number of GABA molecules within 1.0 nm of each ‘random’ sample point is 4.55 ± 1.8. Thus, there does not appear to be a significantly increased population of GABA molecules initially moving towards the midpoint region (6 vs 4.55 ± 1.8). The probability of a GABA molecule randomly reaching the 2.5 nm shell within a specific region of radius 1 nm is ~4.3% (the percentage of available sampling space that is within 1 nm of a specific point, see **Fig 8**). Given that the average distribution we observe is ~4.55%, the initial movement of the GABA molecules is indeed almost completely random/non-biased in nature.

**Fig 9:**
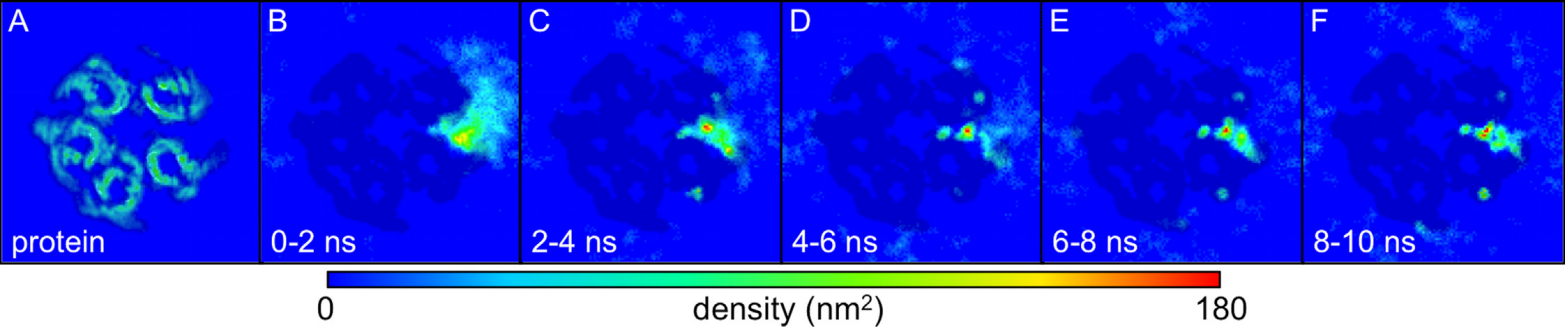
GABA density in horizontal slice through the system. (A) The protein density in an XY slice through the simulation box at a set Z-axis position. This protein density is averaged over the duration of several randomly selected simulations and between 8–9 nm on the Z-axis. (B-F) The densities of GABA molecules from all 100 simulations are averaged over the same slice are shown over time. The ‘shadow’ of the protein density is represented to illustrate the relative position of the GABA density to the protein. Panel B shows the 100 GABA molecules starting at the side of the protein. Panels C-D show the GABA molecules beginning to “bind” in the binding site with others dispersing away from the binding site. Finally, Panels E-F show the highest density of GABA molecules at the binding site (red) and some others distributed all around the protein.

However, once the GABA molecules ‘randomly’ reach the vicinity of the midpoint, the influence of the electrostatic field takes over, and the molecules are funneled and focused into the binding pocket. This funnel acts as a sink on the population of randomly moving GABA molecules; even though the GABA molecules are unbiased and can reach any point on the 2.5 nm shell. If the GABA molecules reach a point on the 2.5 nm shell that is near the vicinity of the midpoint, the molecule becomes ‘trapped’ and is concentrated into the binding region. The time-dependent density distributions of the GABA molecules further illustrate this hypothesis (**Fig 9 and S5 Fig**). The molecules that arrive at the midpoint/binding site regions persist for substantial periods of time, and thus these areas become more densely occupied by GABA molecules (**Fig 9B–9F**), while the other GABA molecules randomly disperse from their starting locations. The GABA-midpoint distance calculations also support the idea of the focusing of the GABA molecules toward the binding site with 86% (24 of 28) of the simulations successfully proceeding to the binding region once they have reached the midpoint.

### Why do we get three ‘binding’ outcomes?

All of the trajectories that fall into the three different simulation categories (BIND, PARTIAL, and NEARBY) have been thoroughly analyzed. We find that the GABA molecules in these different categories all follow similar pathways, all converge at the same point ~1.9–2.0 nm from the GABA binding site COM, and importantly, all at least *mostly* enter the GABA binding region. However, the question remains, if the pathways are so similar, and they all reach this converged midpoint, then why do we observe these three distinct outcomes?

**Fig 10:**
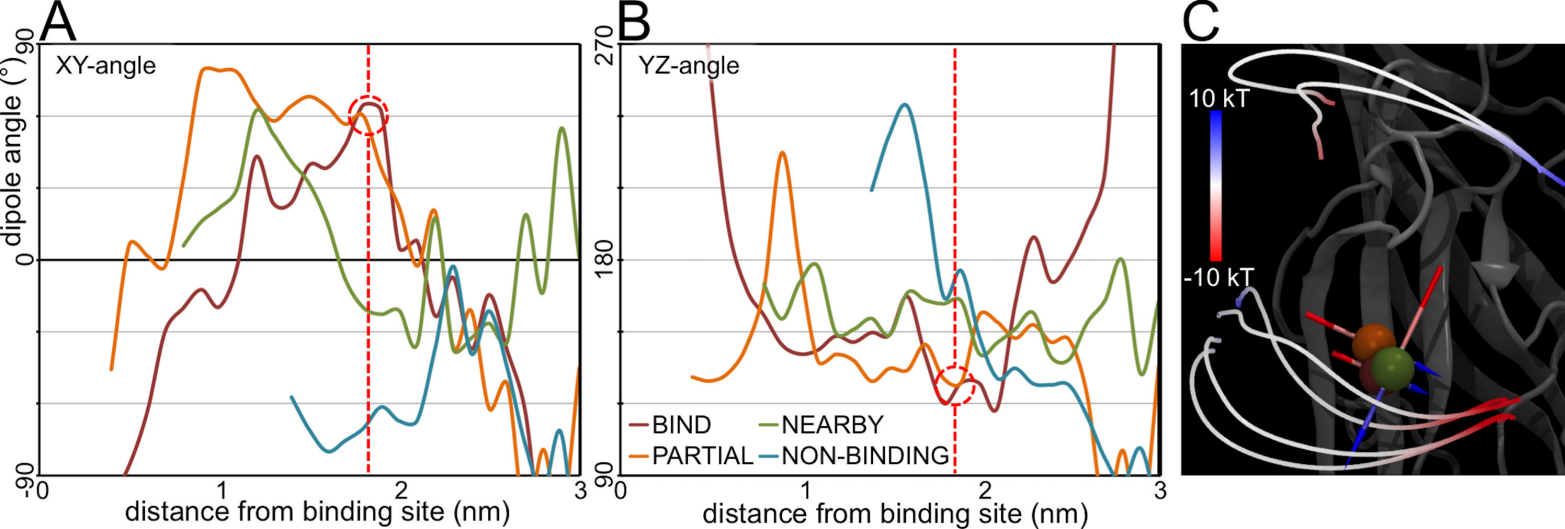
Analysis of the dipole angles. The orientation of the average net dipoles on the GABA molecules is decomposed into the angles the dipole vector makes with the XY plane (A) and the YZ plane (B). The red dashed line indicates the position at which the GABA molecules enter the electrostatic field, with the red dashed circle highlighting the extremely similar values for the BIND and PARTIAL categories. The vectors of these dipoles on the BIND (red), PARTIAL (orange) and NEARBY (green) categories at this position are also represented visually (C).

The orientation of the net dipole on the GABA molecules was decomposed into two components; the angle it makes with the XY plane, and the angle it makes with the YZ plane (**Fig 10**). When the molecules reach the midpoint at 1.9 nm from the binding site COM, the orientations of the dipole in both the BIND and PARTIAL categories are virtually identical (angles of ~65° and ~60°, respectively). However, the orientation of the dipole in the NEARBY category is nearly orthogonal to the BIND and PARTIAL dipoles (**Fig 10C**). Thus, the GABA molecules in the NEARBY category enter the electrostatic field with a different orientation.

**Fig 11:**
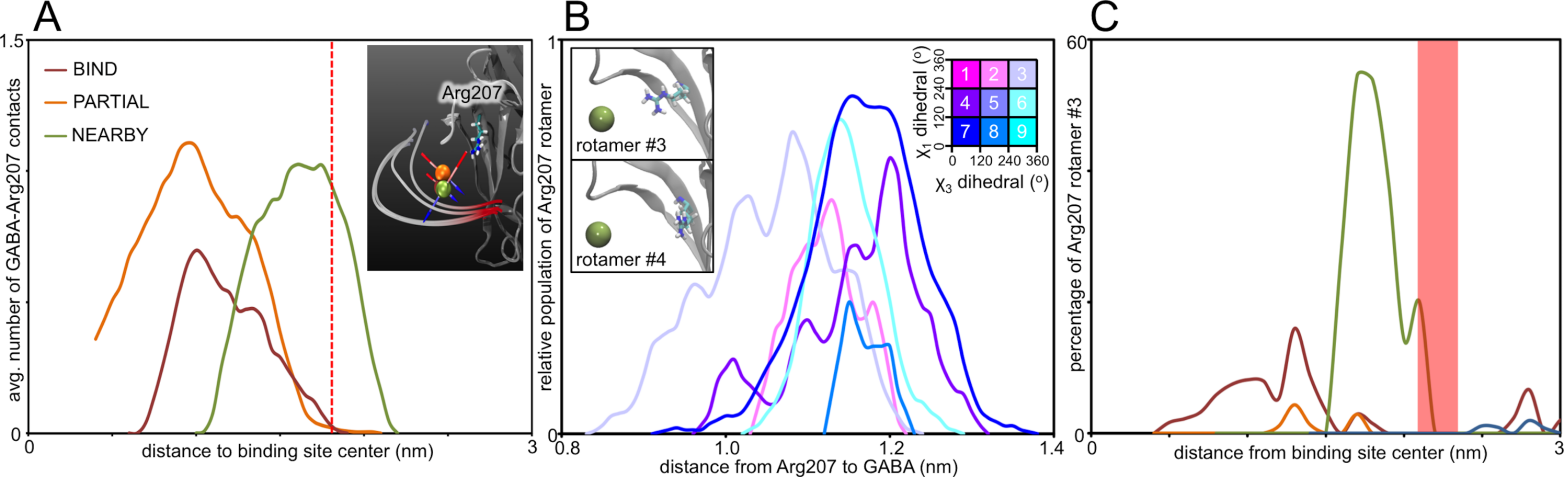
Contacts between GABA and Arg207. (A) The average number of contacts between GABA and Arg207 are shown for the BIND (red), PARTIAL (orange) and NEARBY (green) categories as a function of their distance from the binding site COM. The red dashed line indicates the distance at which the GABA molecules enter the electrostatic field. This is visually depicted (A–inset), showing the net dipoles on the GABA molecules relative to the position of Arg207. (B) Analysis of Arg207 rotamers. The nine possible rotamers formed by the N-CA-CB-CG (χ_1_ dihedral) and CB-CG-CD-NE (χ_1_ dihedral) dihedrals of Arg207 were analyzed in terms of their distance to the average GABA position (for the bin when GABA is 2 nm from the binding site). These distances were calculated and standardized for each rotamer during all of the NEARBY simulations. This shows that the conformation of rotamer #3 allows Arg207 to get significantly closer to GABA than any other rotamer. This is illustrated by a visual comparison between rotamer #3 and rotamer #4 (B–inset, where the position of GABA is represented by an olive green sphere). (C) The percentage population of Arg207 rotamers for each simulation category was calculated as function of the GABA distance from the binding site. This shows that in the NEARBY simulations, Arg207 occupies rotamer #3 for a significant percentage of time as GABA is entering the electrostatic field (indicated by the highlighted red region), whereas in the BIND and PARTIAL simulations Arg207 does not occupy this rotameric conformation at all during the time GABA is entering the electrostatic field.

One reason for this alternate orientation is the presence of a charged amino acid sidechain near the GABA molecule. The Arg207 residue from the β_3_ subunit is close to the midpoint and may influence the GABA alignment. Visual assessment agrees with this hypothesis (**Fig 11A, inset**). Furthermore, analysis of the number of contacts that Arg207 makes to GABA indicates that prior to the midpoint, GABA-Arg207 contacts are only present in the NEARBY simulations (**Fig 11A**). A possible cause for these ‘premature’ GABA-Arg207 contacts is revealed in the distribution of arginine sidechain rotamers (**Fig 11B**). Of the nine possible rotamers formed by the N-CA-CB-CG and CB-CG-CD-NE dihedrals, only one allows consistent positioning to a location where GABA contacts are possible (**Rotamer #3, Fig 11B,** where the N-CA-CB-CG and CB-CG-CD-NE dihedrals are both between 240° and 360°). This rotamer accounts for a significant (~20%) proportion of Arg207 conformations as GABA enters the electrostatic field during the NEARBY simulations (**Fig 11C**), but is not present at the equivalent position in any of the BIND and PARTIAL simulations.

The increased population of this specific arginine rotamer in the NEARBY simulations may form additional contacts to GABA molecules, which subsequently influences their orientation as they enter the electrostatic field. Thus, the GABA molecules in the NEARBY simulations are in a different orientation and appear to be ‘swept along’ to a slightly different position, leaving them in a sub-optimal orientation to then ‘flip up’ into the binding pocket. Notably, most of the GABA molecules in this category actually persist near, or just inside the binding region for the duration of the simulations, and it may be the case that given extended simulation time they would indeed eventually reorient into the GABA binding site. Our statistical analysis does not reveal the cause-and-effect relationship between the Arg207 rotamer and the GABA orientation. We have hypothesized that the possible increased presence of Arg207 rotamer #3 induces the altered orientation of the GABA molecule. However, it is also plausible that the GABA molecule may have already adopted that orientation, and it is the occurrence of this orientation that causes the increase in the Arg207 rotamer #3 population.

Therefore, a differing orientation may account for the varying behavior seen in the NEARBY category of simulations. In contrast, the BIND and PARTIAL simulations both occupy the ‘correct’ orientation and as such, both proceed into the binding site. Once in the binding site, the GABA molecules from the BIND trajectories remain there for the remainder of the simulation (an average of almost 7 ns—70% of the simulation time), whereas molecules from the PARTIAL trajectories leave after an average of only ~2.4 ns.

To suggest a potential basis for this differing behavior, components of the binding site were investigated. As the system was fully solvated and had an effective ionic concentration of 0.15 M, there are 135 Cl^-^ ions present in the simulation. Analysis of the Cl^-^ density shows that during the PARTIAL simulations (where GABA leaves the binding pocket), there is a higher density of Cl^-^ ions in the core of the binding pocket compared to the BIND simulations. Early GABA_A_-R studies showed that higher concentrations of Cl^-^ ions modulate GABA binding [35, 36], as well as inhibiting muscimol binding into the GABA site [37]. Moreover, anions in general have been reported to perturb GABA binding [38, 39], suggesting that they may prevent/shield key interactions between the carboxyl end of the GABA molecule and the basic binding residues.

## Discussion

Through unbiased molecular dynamics simulations we have constructed a possible pathway by which GABA molecules enter the binding site of the GABA receptor. We also hypothesize an electrostatically driven mechanism for these GABA molecules to be ‘detected’. The GABA_A_-R system represents an ideal test case to investigate ligand binding, as the binding site is well defined and the ligand interactions are primarily electrostatic, which have long-range effects. This methodology may be applicable to other protein systems in terms of characterization of the ligand-binding pathway.

Our findings indicate that all the GABA molecules that at least partly enter the binding pocket follow a very similar pathway, whereby they approach the protein from below the binding site, before progressing up behind the C-loop and into the binding pocket. The pathway passes through a ‘midpoint’. This midpoint is a region ~1 nm below the binding site, and ~1 nm laterally out away from the binding site, where the combined electrostatic potential surface of the protein creates a very strong electrostatic field.

The midpoint is a critical decision point, where the GABA molecule is ‘captured’ by the receptor and its far-reaching electrostatic field. The behavior and distribution of the GABA molecules up to this point is almost random/unbiased, as illustrated by their indiscriminate initial dispersion (**Fig 8**) and distribution density (**S5 Fig**). However, once the GABA molecules reach the midpoint they get ‘swept along’ by the electrostatic field, which funnels them to a position much nearer the binding site, whereupon more specific (but shorter distance) interactions can take over. The ‘capturing’ nature of the midpoint is highlighted by the fact that 86% (24 of 28) of the simulations where GABA reaches the midpoint then proceed on to the binding region. This is further illustrated by accumulating density of GABA molecules in this area (**Fig 9 and S5 Fig**).

Thus, the binding pathway may operate in a two-step, two-resolution manner. The longer-range electrostatic field interactions are used to ‘pull in’ molecules that match the general properties of the GABA_A_-R agonist–small and zwitterionic. Once these molecules have been brought closer to the protein, the precise sidechain-ligand contacts could determine if the molecule is suitable for binding. Previous studies suggest that the electrostatic interactions between glutamate and the glutamate receptor LBD become significant at ~5 Å [40]. Thus, diffusing glutamates within about 5 Å of the protein are readily drawn in to the binding site through electrostatic interactions. We suggest that the effects on GABA may be significant at even longer distance.

This hypothesis of a non-specific electrostatic interaction is further highlighted by the fact that the electronegativity is a general property of the region, rather than a specific residue, and that the midpoint coordinates are actually ~0.6 nm from the surface of the protein. Experimental investigation into the residues of this region may require more than single-point mutagenesis in order to alter the overall electronegative nature of the area. A fundamental difficulty in the assessment of mutation effects is that if ion flow through the channel is the endpoint measurement, then it can be problematic to distinguish a change in ligand binding versus a change in channel gating.

## Methods

### Model construction and validation

The model used in this work was derived from previously published studies, [21, 32] which contain more detailed method descriptions. In brief, the main template of the GABA_A_-R model was the Torpedo marmorata nAChR structure (PDB [41] ID: 2BG9). [42] This template was used as a scaffold upon which other better-resolution, higher-homology protein sections were incorporated. This protocol was used for regions that were missing in the structure, lacked alignment to the template, or were poorly conserved. The DIG (Deletions Insertions Gaps) tool from the LGA (Local–Global Alignment) [43] program was used for this additional alignment and modeling. The DIG tool analyzes protein structures, or fragments of protein structures, and can complete the missing sections by searching for areas of similar sequence from a database of structural folds or a manually selected library of appropriate structural regions. In this study, as we were particularly interested in GABA binding, we concentrated on the LBD. Thus, we focused on gaps and regions that were poorly modeled/aligned in this domain. In order to fill these gaps and increase the accuracy of these regions, we searched homologous sections from all available pLGIC structures and the LBD-analogous AChBPs (such as PDB IDs: 2BYN [23] and 1UX2 [44]). Thus, the models of the LBD domains were completed and refined using these additional structural data, resulting in an overall model that has a better resolution and is more closely aligned to the GABA_A_-R sequence. In order to increase the likelihood of observing a GABA-binding event, the starting LBD structure was modeled using the available apo structures, rather than structures with a ligand-bound, ‘closed’ C-loop conformation, such as the glutamate-bound GluCl [34]. We also chose to model the α_6_β_3_δ receptor as it has the highest affinity [8] for GABA and thus the greatest chance of binding success. Consistent with published modeling studies of GABA_A_-R, [33, 45] the cytoplasmic domain was not included due to high structural uncertainty. The integrity of these models was assessed using PROCHECK [46, 47]. Most parameters were typical of a structure of 1.5–2.5 Å resolution, an improvement on the main template resolution, and an enhancement of the overall quality.

The protein model was inserted into a preformed and equilibrated POPC (palmitoyl-oleoyl phosphatidylcholine) bilayer that was used for previous GABA_A_-R simulations. [21, 33] The system was solvated and had counter-ions added to neutralize the charge. Further Na+/Cl− ions were added to create an effective concentration of 0.15 M. The final system (consisting of ~210,000 atoms) was energy minimized. Following minimization, the system was simulated for 2 ns with harmonic positional restraints on the protein, allowing the relaxation of lipid and water molecules. After allowing the packing of the lipids around the protein, the area per lipid was calculated and found to be representative of POPC. Subsequently, the area of the XY-plane was fixed (to maintain the area per lipid), positional restraints were removed, and the entire apo system was run for a further 20 ns to fully equilibrate the system. The root-mean-square deviation (RMSD) of the Cα atoms during this equilibration compared favorably to previous GABA_A_-R simulations [33]. DSSP (Define Secondary Structure of Proteins) [48] was used to measure the secondary-structure conformation of the GABA_A_-R. This confirmed that the structural composition of the GABA_A_-R model remained consistent throughout the 20 ns equilibration. Finally, there are critical GABA_A_-R salt bridges that are known to be important for gating dynamics [49] or maintaining the binding pocket structure [19, 25]. Analysis showed that these salt bridges were present in the homology model and remained intact during equilibration.

### Simulation system setup

**Fig 12:**
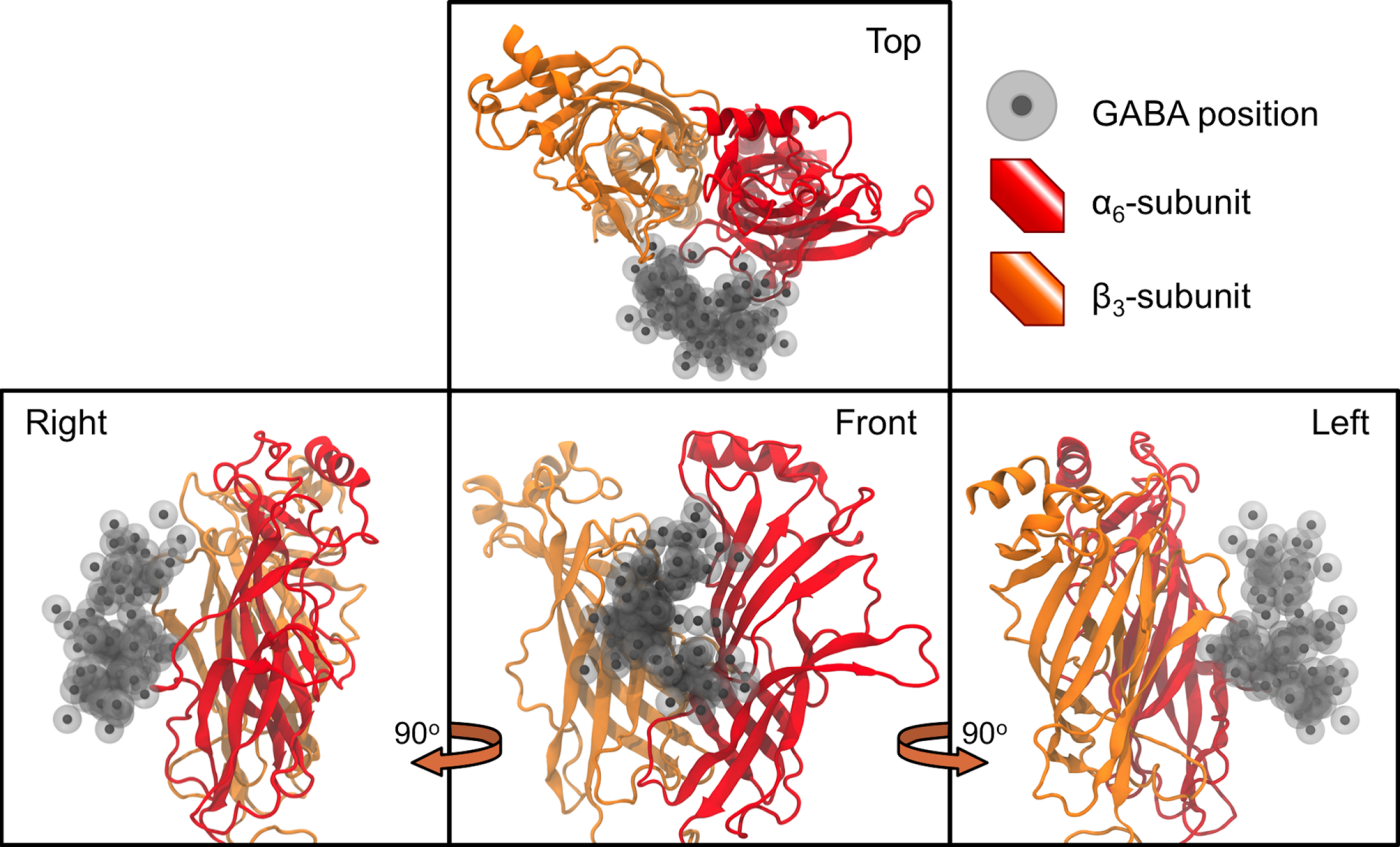
The random starting positions of the GABA molecules. The initial starting locations of GABA in each of the 100 simulations are superimposed to show their positions relative to the GABA_A_-R. The ligand binding domains of the α_6_-subunit (red) and β_3_-subunit (orange) are also represented.

Four separate, random frames were taken from the last 5 ns of the apo GABA_A_-R equilibration simulation. In each of these four frames, a GABA molecule was placed in a random orientation at a different location **~2.50 nm** from the center of the binding site. These four systems were used as ‘seed points’ and each underwent molecular dynamics simulations for one ns. From each of these four seed simulations, 25 frames (output at every 2 ps) were randomly chosen, producing 100 different starting positions for GABA in various conformations and orientations that are **~2.46 ± 0.50 nm** from the center of the binding site **(Figs 2 and 12)**. Thus, unbiased, randomly oriented starting positions for GABA molecules were generated. Okada *et al*. [50] recently demonstrated that they could achieve binding of a ligand within ~2 ns when it was placed ~0.5 nm from the binding site. Thus, for our systems with a starting distance of ~2.5 nm to the center of the binding site, a timescale of 10 ns was an appropriate choice to observe binding. Thus, each of the 100 systems was simulated for 10 ns, to produce 1 μs of total data for subsequent analysis.

All molecular dynamics (MD) simulations were run using CHARMM [51] (v27 for lipid and protein) in NAMD [52]. As GABA is a simple amino acid, we used the PARAtool [53] VMD [54] plugin to carry out force field parameterization by analogy to existing residues in the CHARMM force field to determine the bonded interactions. The partial charges are obtained using the RESP procedure with GAUSSIAN03 [55], at the Hartree-Fock 6-31G* [56–58] level of theory. GABA was modeled as a zwitterionic molecule. All systems use NPT and a nonbonded vdw cutoff of 1.2 nm. Constant pressure and temperature were maintained with Langevin pistons set at 1 atm and 310 K, using an oscillation time constant of 200 fs, a damping time constant of 50 fs, and a temperature damping coefficient of 1/ps. As the CHARMM force field parameters used do not reproduce the observed area per lipid over long MD trajectories, the area of the system in the XY-plane was kept constant. All simulations were run with particle mesh Ewald electrostatics, SHAKE [59], and TIP3P water [60], and with a 2 fs time step. All the simulations were run on the Sierra Linux cluster at the Livermore Computing Center (LC), consisting of 23,328 Intel Xeon EP X5660 cores.

### Trajectory preparation

After the completion of the simulations, the trajectories were categorized depending on the distance of GABA relative to the binding site. As we were investigating the *binding* pathway, further preparation was needed for some of the simulations. In all of the **PARTIAL** and some of the **NEARBY** trajectories, the GABA molecules reach a position within or near the binding site COM, but subsequently leave and diffuse away. These ‘leaving’ sections of the simulation may obscure the analysis results and were not of interest. In those instances, the trajectories are split at the point where the GABA molecule reaches its minimum distance to the binding site COM. The second ‘leaving’ section of the trajectory was discarded, and only the first ‘binding’ section was used for analysis.

After refining the simulations by removing the ‘leaving’ sections, all the trajectories within a particular category were combined. In order to standardize the molecule positions and orientations, the frames (output at every 20 ps) within each group were aligned to the Cα atoms of the protein backbone by a least-squares fitting method. These frames were sorted based upon the distance between the GABA COM and the binding site COM. These sorted frames were binned into windows defined by 0.1 nm increments of this GABA COM to binding site COM distance. The overall average number of descriptors (such as position, standard deviation of position, dipole direction, and dipole strength) of the GABA molecules within these distance-defined bins were calculated for each bin and compared between the four categories; **BIND**, **PARTIAL**, **NEARBY**, and **RANDOM**.

While we have defined a GABA molecule as ‘binding’ when within a distance cutoff to the binding site COM, these compounds still may not occupy the correct binding orientation. Indeed, the objective of this study is to determine the route and mechanism by which the GABA molecules get into the binding pocket, but not the specific details of sidechain contacts or subtle molecule rearrangements once within the pocket. In fact, formation of these precise interactions may be beyond the timescale of our study.

**Fig 13:**
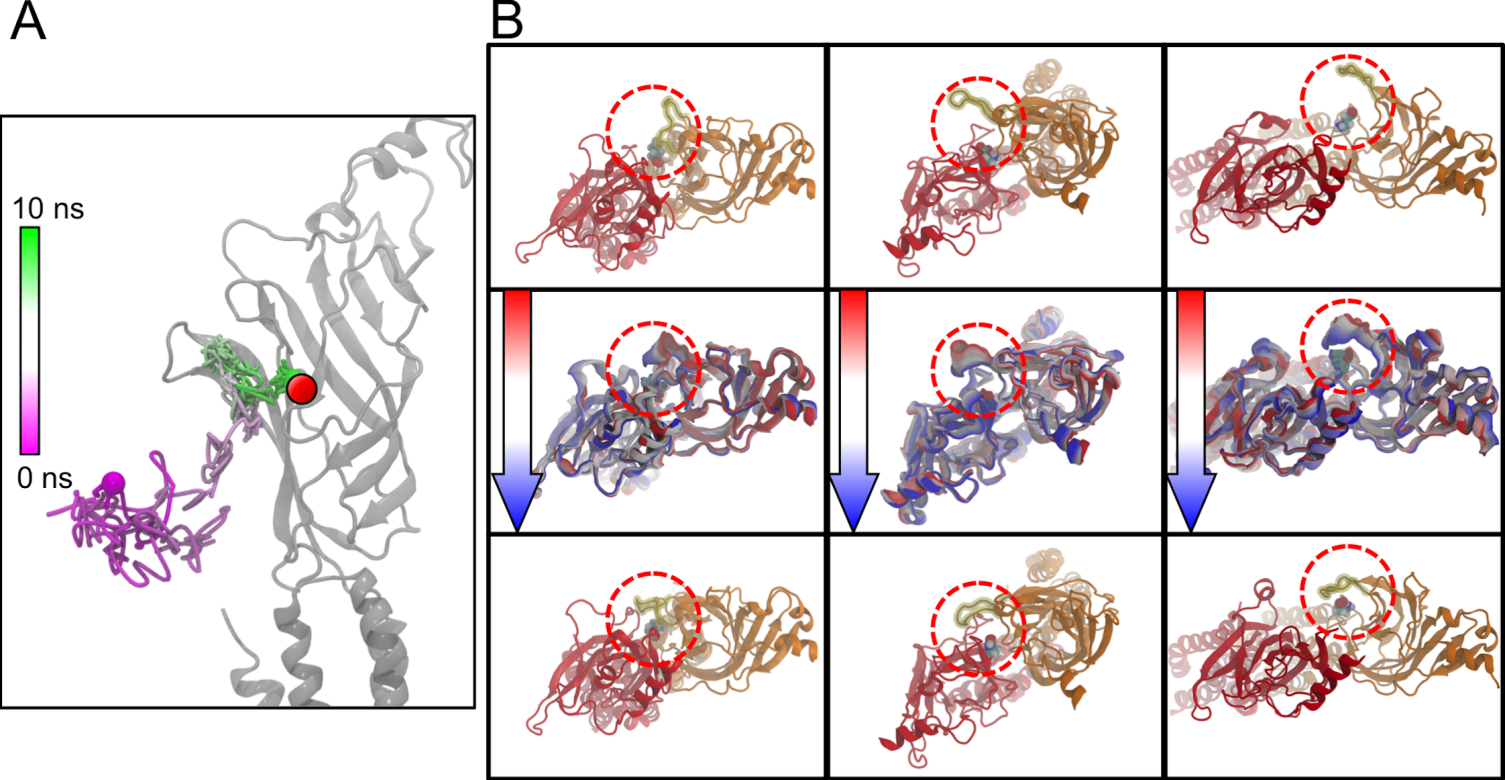
Observed closure of the C-loop. (A) The complete pathway of one of the BIND simulations is shown, plotting the position of the GABA molecule from the start of the simulation (magenta) to the end (green). The binding site is illustrated by the red circle on the grey-colored β_3_-subunit. (B) Three of the BIND simulations displayed closure of the C-loop over the bound GABA molecule. In the top and bottom rows, the β_3_-subunit and α_6_-subunit of the binding site are shown from a top view in orange and red, respectively, with the C-loop highlighted by the dashed red circle. The top row shows the C-loop in an open conformation, while the bottom row shows the C-loop in a closed conformation. The middle row illustrates the closing pathway of the loop starting from the open (red) conformation, and moving through white to the closed (blue) conformation. Each of the simulations is portrayed from a slightly different viewpoint in order to most easily see the C-loop movement.

Despite this, however, we are confident that our ‘binding’ simulations do indeed represent the initial stages of true GABA binding. The BIND simulations reach a final position that almost overlaps the binding site COM (**Fig 13A**), and once they have reached that position, they persist there for the remainder of the simulation (only undergoing minor fluctuations in position with the binding site; as possible indication of GABA reorientation into an optimal binding conformation). Furthermore, in three of the nine BIND simulations, after GABA has reached the binding site, closure of the C-loop around the GABA molecule was observed (**Fig 13B**). This process is indicative of the early stages of LGIC activation by a ligand and may represent GABA reaching the appropriate binding site position.

### Analysis and visualization

The PDB2PQR [61, 62] and Adaptive Poisson-Boltzmann Solver [63–65] software packages were used for the modeling of the biomolecular solvation through the solution of the Poisson-Boltzmann equation. VMD [52, 54] was used to map the electrostatic potential to the biomolecular surface to produce an electrostatic potential surface and visualization of the electrostatic field lines. All further analysis was carried out using GROMACS [66] functions, VMD, and locally written scripts. Figure preparation was done using VMD.

## Supporting Information

S1 Fig The initial movements of the GABA molecules. A histogram depicts the ratios of the GABA to binding site distance after 1 (black line), 50 (red line), and 100 (green line) ps compared to the initial GABA to binding site distance for all 100 simulations.
(TIFF)

S2 Fig Electrostatic surfaces of alternative GABA_A_-R isoforms and homology models. The electrostatic surfaces were calculated for various different GABA_A_-R homology models that were constructed using either different GABA_A_-R subunit sequences or different structural templates. They are (L-R): model of the alternative α_1_β_2_γ_2_ isoform, α_6_β_3_δ model using a complete AChBP for the LBD template, α_6_β_3_δ model using GluCl as a template, α_6_β_3_δ model using the new GABA_A_-R β_3_ homopentamer as a template.
(TIFF)

S3 Fig Properties of the GABA population deviate ~1.9–2.0 nm from the GABA binding site. The standard deviation of the population of GABA molecules (black) and the average dipole of the GABA population (grey) both show a distinct change in behavior ~1.9–2.0 nm from the binding site COM (indicated by the dashed black line). These values were calculated using all of the binding simulations (BIND, PARTIAL, and NEARBY).
(TIFF)

S4 Fig GABA distance to the midpoint. Of the 100 independent simulations, GABA accesses the midpoint area (defined as reaching a point < 1 nm away) in 28 of them (A), leaving 72 simulations where GABA does not reach the vicinity of the midpoint at all (B).
(TIFF)

S5 Fig GABA density in vertical slices through the system. The system was divided into 1 nm thick slices starting from the front of the system and ending at the middle of the protein. The depth of the slices are indicated by a rainbow color scale. The protein density is averaged over the duration of several randomly selected simulations. The density of GABA molecules from all 100 simulations averaged over the same slice, shown as time progresses. The vertical red line through the 3–4 nm slices indicates the center of the GABA binding site.
(TIFF)

## References

[1] Tretter V, Ehya N, Fuchs K, Sieghart W. Stoichiometry and assembly of a recombinant GABAA receptor subtype. J Neurosci. 1997;17(8):2728–37. . 9092594 10.1523/JNEUROSCI.17-08-02728.1997 PMC6573102

[2] Baumann SW, Baur R, Sigel E. Forced subunit assembly in alpha1beta2gamma2 GABAA receptors. Insight into the absolute arrangement. J Biol Chem. 2002;277(48):46020–5. . 12324466 10.1074/jbc.M207663200

[3] Nusser Z, Mody I. Selective modulation of tonic and phasic inhibitions in dentate gyrus granule cells. J Neurophysiol. 2002;87(5):2624–8. . 11976398 10.1152/jn.2002.87.5.2624

[4] Nusser Z, Sieghart W, Somogyi P. Segregation of different GABAA receptors to synaptic and extrasynaptic membranes of cerebellar granule cells. J Neurosci. 1998;18(5):1693–703. . 9464994 10.1523/JNEUROSCI.18-05-01693.1998 PMC6792611

[5] Wei W, Zhang N, Peng Z, Houser CR, Mody I. Perisynaptic localization of delta subunit-containing GABA(A) receptors and their activation by GABA spillover in the mouse dentate gyrus. J Neurosci. 2003;23(33):10650–61. . 14627650 10.1523/JNEUROSCI.23-33-10650.2003 PMC6740905

[6] Farrant M, Nusser Z. Variations on an inhibitory theme: phasic and tonic activation of GABA(A) receptors. Nat Rev Neurosci. 2005;6(3):215–29. . 15738957 10.1038/nrn1625

[7] Mody I. Distinguishing between GABA(A) receptors responsible for tonic and phasic conductances. Neurochem Res. 2001;26(8–9):907–13. . 11699942 10.1023/a:1012376215967

[8] Storustovu SI, Ebert B. Pharmacological characterization of agonists at delta-containing GABAA receptors: Functional selectivity for extrasynaptic receptors is dependent on the absence of gamma2. J Pharmacol Exp Ther. 2006;316(3):1351–9. Epub 2005/11/08. doi: jpet.105.092403 [pii] 10.1124/jpet.105.092403 . 16272218

[9] Miller PS, Aricescu AR. Crystal structure of a human GABAA receptor. Nature. 2014;512(7514):270–5. Epub 2014/06/10. 10.1038/nature13293 . 24909990 PMC4167603

[10] Mokrab Y, Bavro VN, Mizuguchi K, Todorov NP, Martin IL, Dunn SM, et al Exploring ligand recognition and ion flow in comparative models of the human GABA type A receptor. J Mol Graph Model. 2007;26(4):760–74. . 17544304 10.1016/j.jmgm.2007.04.012

[11] Ernst M, Bruckner S, Boresch S, Sieghart W. Comparative models of GABAA receptor extracellular and transmembrane domains: important insights in pharmacology and function. Mol Pharmacol. 2005;68(5):1291–300. . 16103045 10.1124/mol.105.015982

[12] Smith GB, Olsen RW. Functional domains of GABAA receptors. Trends Pharmacol Sci. 1995;16(5):162–8. . 7624971 10.1016/s0165-6147(00)89009-4

[13] Ernst M, Brauchart D, Boresch S, Sieghart W. Comparative modeling of GABA(A) receptors: limits, insights, future developments. Neuroscience. 2003;119(4):933–43. . 12831854 10.1016/s0306-4522(03)00288-4

[14] Wagner DA, Czajkowski C. Structure and dynamics of the GABA binding pocket: A narrowing cleft that constricts during activation. J Neurosci. 2001;21(1):67–74. . 11150321 10.1523/JNEUROSCI.21-01-00067.2001 PMC6762441

[15] Boileau AJ, Newell JG, Czajkowski C. GABA(A) receptor beta 2 Tyr97 and Leu99 line the GABA-binding site. Insights into mechanisms of agonist and antagonist actions. J Biol Chem. 2002;277(4):2931–7. . 11711541 10.1074/jbc.M109334200

[16] Holden J, Czajkowski C. alpha1Gly124-alpha1Leu132: a novel binding site region on the GABAA receptor that undergoes distinct conformational rearrangements during ligand binding and allosteric modulation. Soc Neurosci Abstr. 2004;34:(50.1).

[17] Newell JG, McDevitt RA, Czajkowski C. Mutation of glutamate 155 of the GABAA receptor beta2 subunit produces a spontaneously open channel: a trigger for channel activation. J Neurosci. 2004;24(50):11226–35. . 15601928 10.1523/JNEUROSCI.3746-04.2004 PMC6730373

[18] Wagner DA, Czajkowski C, Jones MV. An arginine involved in GABA binding and unbinding but not gating of the GABA(A) receptor. J Neurosci. 2004;24(11):2733–41. . 15028766 10.1523/JNEUROSCI.4316-03.2004 PMC6729509

[19] Venkatachalan SP, Czajkowski C. A conserved salt bridge critical for GABA(A) receptor function and loop C dynamics. Proc Natl Acad Sci USA. 2008;105(36):13604–9. 10.1073/pnas.0801854105 18757734 PMC2533236

[20] Smith GB, Olsen RW. Identification of a [3H]muscimol photoaffinity substrate in the bovine gamma-aminobutyric acidA receptor alpha subunit. J Biol Chem. 1994;269(32):20380–7. . 8051133

[21] Carpenter TS, Lau EY, Lightstone FC. A Role for Loop F in Modulating GABA Binding Affinity in the GABAA Receptor. J Mol Biol. 2012;422(2):310–23. 10.1016/j.jmb.2012.05.025 22659322

[22] Chang YC, Wu W, Zhang JL, Huang Y. Allosteric activation mechanism of the cys-loop receptors. Acta Pharmacol Sin. 2009;30(6):663–72. Epub 2009/05/16. 10.1038/aps.2009.51 . 19444220 PMC4002373

[23] Hansen SB, Sulzenbacher G, Huxford T, Marchot P, Taylor P, Bourne Y. Structures of Aplysia AChBP complexes with nicotinic agonists and antagonists reveal distinctive binding interfaces and conformations. EMBO Journal. 2005;24(20):3635–46. 10.1038/Sj.Emboj.7600828 . 16193063 PMC1276711

[24] Celie PH, Klaassen RV, van Rossum-Fikkert SE, van Elk R, van Nierop P, Smit AB, et al Crystal structure of acetylcholine-binding protein from Bulinus truncatus reveals the conserved structural scaffold and sites of variation in nicotinic acetylcholine receptors. J Biol Chem. 2005;280(28):26457–66. Epub 2005/05/19. doi: M414476200 [pii] 10.1074/jbc.M414476200 . 15899893

[25] Mukhtasimova N, Free C, Sine SM. Initial coupling of binding to gating mediated by conserved residues in the muscle nicotinic receptor. J Gen Physiol. 2005;126(1):23–39. . 15955875 10.1085/jgp.200509283 PMC2266616

[26] Auerbach A. The gating isomerization of neuromuscular acetylcholine receptors. J Physiol. 2010;588(Pt 4):573–86. Epub 2009/11/26. 10.1113/jphysiol.2009.182774 19933754 PMC2828132

[27] Belfield WJ, Cole DJ, Martin IL, Payne MC, Chau PL. Constrained geometric simulation of the nicotinic acetylcholine receptor. J Mol Graph Model. 2014;52:1–10. 10.1016/j.jmgm.2014.05.001 . 24955489

[28] Zheng W, Auerbach A. Decrypting the sequence of structural events during the gating transition of pentameric ligand-gated ion channels based on an interpolated elastic network model. PLoS Comput Biol. 2011;7(1):e1001046 Epub 2011/01/22. 10.1371/journal.pcbi.1001046 21253563 PMC3017109

[29] Calimet N, Simoes M, Changeux JP, Karplus M, Taly A, Cecchini M. A gating mechanism of pentameric ligand-gated ion channels. Proc Natl Acad Sci USA. 2013;110(42):E3987–96. Epub 2013/09/18. 10.1073/pnas.1313785110 24043807 PMC3801054

[30] Wlodek ST, Shen T, McCammon JA. Electrostatic steering of substrate to acetylcholinesterase: analysis of field fluctuations. Biopolymers. 2000;53(3):265–71. Epub 2000/02/19. 10.1002/(SICI)1097-0282(200003)53:3<265::AID-BIP6>3.0.CO;2-N . 10679631

[31] Tan RC, Truong TN, McCammon JA, Sussman JL. Acetylcholinesterase: electrostatic steering increases the rate of ligand binding. Biochemistry. 1993;32(2):401–3. Epub 1993/01/19. . 8422348 10.1021/bi00053a003

[32] Carpenter TS, Lau EY, Lightstone FC. Identification of a possible secondary picrotoxin-binding site on the GABA(A) receptor. Chem Res Toxicol. 2013;26(10):1444–54. Epub 2013/09/14. 10.1021/tx400167b . 24028067

[33] Law RJ, Lightstone FC. Modeling neuronal nicotinic and GABA receptors: important interface salt-links and protein dynamics. Biophys J. 2009;97(6):1586–94. Epub 2009/09/16. doi: S0006-3495(09)01220-X [pii] 10.1016/j.bpj.2009.06.044 19751663 PMC2749782

[34] Hibbs RE, Gouaux E. Principles of activation and permeation in an anion-selective Cys-loop receptor. Nature. 2011;474(7349):54–60. Epub 2011/05/17. doi: nature10139 [pii] 10.1038/nature10139 . 21572436 PMC3160419

[35] Madtes P Jr. Chloride ions preferentially mask high-affinity GABA binding sites. J Neurochem. 1984;43(5):1434–7. Epub 1984/11/01. . 6092539 10.1111/j.1471-4159.1984.tb05405.x

[36] Shank RP, Baldy WJ, Mattucci LC, Villani FJ Jr. Ion and temperature effects on the binding of gamma-aminobutyrate to its receptors and the high-affinity transport system. J Neurochem. 1990;54(6):2007–15. Epub 1990/06/01. . 2159983 10.1111/j.1471-4159.1990.tb04905.x

[37] Matsumoto K, Fukuda H. Anisatin modulation of temperature-dependent inhibition [3H]muscimol binding by chloride ion. Brain Res. 1983;270(1):103–8. Epub 1983/06/27. . 6307484 10.1016/0006-8993(83)90795-3

[38] Enna SJ, Snyder SH. Influences ions, enzymes, and detergents on gamma-aminobutyric acid-receptor binding in synaptic membranes of rat brain. Mol Pharmacol. 1977;13(3):442–53. Epub 1977/05/01. . 876033

[39] Browner M, Ferkany JW, Enna SJ. Biochemical identification of pharmacologically and functionally distinct GABA receptors in rat brain. J Neurosci. 1981;1(5):514–8. Epub 1981/05/01. . 7346566 10.1523/JNEUROSCI.01-05-00514.1981 PMC6564165

[40] Odai K, Sugimoto T, Kubo M, Ito E. Theoretical research on structures of gamma-aminobutyric acid and glutamic acid in aqueous conditions. Journal of biochemistry. 2003;133(3):335–42. Epub 2003/05/23. . 12761169 10.1093/jb/mvg045

[41] Berman H, Westbrook J., Feng Z., Gilliland G., Bhat T.N., Weissig H., et al The Protein Data Bank. Nucleic Acids Res. 2000;28:235–42. 10592235 10.1093/nar/28.1.235 PMC102472

[42] Unwin N. Refined structure of the nicotinic acetylcholine receptor at 4A resolution. J Mol Biol. 2005;346(4):967–89. . 15701510 10.1016/j.jmb.2004.12.031

[43] Zemla A. LGA: A method for finding 3D similarities in protein structures. Nucleic Acids Res. 2003;31(13):3370–4. Epub 2003/06/26. 12824330 10.1093/nar/gkg571 PMC168977

[44] Celie PH, van Rossum-Fikkert SE, van Dijk WJ, Brejc K, Smit AB, Sixma TK. Nicotine and carbamylcholine binding to nicotinic acetylcholine receptors as studied in AChBP crystal structures. Neuron. 2004;41(6):907–14. . 15046723 10.1016/s0896-6273(04)00115-1

[45] Law RJ, Lightstone FC. GABA receptor insecticide non-competitive antagonists may bind at allosteric modulator sites. Int J Neurosci. 2008;118(5):705–34. Epub 2008/05/01. doi: 792757998 [pii] 10.1080/00207450701750216 . 18446586

[46] Laskowski RA, Macarthur MW, Moss DS, Thornton JM. PROCHECK—A program to check the stereochemical quality of protein structures. J Appl Crystall. 1993;26:283–91.

[47] Laskowski RA, Rullmann JAC, MacArthur MW, Kaptein R, Thornton JM. AQUA and PROCHECK-NMR: Programs for checking the quality of protein structures solved by NMR. J Biomol Nmr. 1996;8(4):477–86. . 9008363 10.1007/BF00228148

[48] Kabsch W, Sander C. Dictionary of protein secondary structure: pattern recognition of hydrogen-bonded and geometrical features. Biopolymers. 1983;22(12):2577–637. Epub 1983/12/01. 10.1002/bip.360221211 . 6667333

[49] Lummis SC, Beene DL, Lee LW, Lester HA, Broadhurst RW, Dougherty DA. Cis-trans isomerization at a proline opens the pore of a neurotransmitter-gated ion channel. Nature. 2005;438(7065):248–52. . 16281040 10.1038/nature04130

[50] Okada O, Odai K, Sugimoto T, Ito E. Molecular dynamics simulations for glutamate-binding and cleft-closing processes of the ligand-binding domain of GluR2. Biophys Chem. 2012;162:35–44. Epub 2012/01/31. 10.1016/j.bpc.2011.12.004 . 22284903

[51] MacKerrell AD, Bashford D, Bellott M, Dunbrack RL, Evanseck JD, Field MJ, et al All-atom empirical potential for molecular modeling and dynamics studies of proteins. J Phys Chem B. 1998;102:3586–616. 10.1021/jp973084f 24889800

[52] Phillips JC, Braun R, Wang W, Gumbart J, Tajkhorshid E, Villa E, et al Scalable molecular dynamics with NAMD. J Comput Chem. 2005;26(16):1781–802. . 16222654 10.1002/jcc.20289 PMC2486339

[53] Saam J, Ivanov I, Walther M, Holzhutter HG, Kuhn H. Molecular dioxygen enters the active site of 12/15-lipoxygenase via dynamic oxygen access channels. Proc Natl Acad Sci USA. 2007;104(33):13319–24. Epub 2007/08/07. 10.1073/pnas.0702401104 17675410 PMC1948941

[54] Humphrey W, Dalke A, Schulten K. VMD: Visual molecular dynamics. J Mol Graphics. 1996;14(1):33–8. 10.1016/0263-7855(96)00018-5 8744570

[55] Frisch MJ, Trucks GW, Schlegel HB, Scuseria GE, Robb MA, Cheeseman JR, et al. Gaussian 03, Revision C.02. 2003.

[56] Becke AD. Density-Functional Thermochemistry .3. The Role of Exact Exchange. J Chem Phys. 1993;98(7):5648–52. 10.1063/1.464913 .

[57] Lee CT, Yang WT, Parr RG. Development of the Colle-Salvetti Correlation-Energy Formula into a Functional of the Electron-Density. Phys Rev B. 1988;37(2):785–9. 10.1103/Physrevb.37.785 . 9944570

[58] Perdew JP, Wang Y. Accurate and Simple Analytic Representation of the Electron-Gas Correlation-Energy. Phys Rev B. 1992;45(23):13244–9. 10.1103/Physrevb.45.13244 . 10001404

[59] Ryckaert JP, Ciccotti G, Berendsen HJC. Numerical integration of the Cartesian equations of motion of a system with constraints: molecular dynamics of *n*-alkanes. J Comput Phys. 1977;23:327–41.

[60] Jorgensen WL, Chandresekhar J, Madura JD, Impey RW, Klein ML. Comparison of simple potential functions for simulating liquid water. J Chem Phys. 1983;79:926–35.

[61] Dolinsky TJ, Czodrowski P, Li H, Nielsen JE, Jensen JH, Klebe G, et al PDB2PQR: expanding and upgrading automated preparation of biomolecular structures for molecular simulations. Nucleic Acids Res. 2007;35(Web Server issue):W522–5. Epub 2007/05/10. 10.1093/nar/gkm276 17488841 PMC1933214

[62] Dolinsky TJ, Nielsen JE, McCammon JA, Baker NA. PDB2PQR: an automated pipeline for the setup of Poisson-Boltzmann electrostatics calculations. Nucleic Acids Res. 2004;32(Web Server issue):W665–7. Epub 2004/06/25. 10.1093/nar/gkh381 15215472 PMC441519

[63] Holst M, Saied F. Multigrid Solution of the Poisson-Boltzmann Equation. J Comput Chem. 1993;14(1):105–13. 10.1002/Jcc.540140114 .

[64] Holst MJ, Saied F. Numerical-Solution of the Nonlinear Poisson-Boltzmann Equation—Developing More Robust and Efficient Methods. J Comput Chem. 1995;16(3):337–64. 10.1002/Jcc.540160308 .

[65] Baker NA, Sept D, Joseph S, Holst MJ, McCammon JA. Electrostatics of nanosystems: application to microtubules and the ribosome. Proc Natl Acad Sci USA. 2001;98(18):10037–41. Epub 2001/08/23. 10.1073/pnas.181342398 11517324 PMC56910

[66] Hess B, Kutzner C, van der Spoel D, Lindahl E. GROMACS 4: Algorithms for highly efficient, load-balanced, and scalable molecular simulation. J Chem Theory Comput. 2008;4(3):435–47. 10.1021/Ct700301q . 26620784

