# Supplementary figures and images for: An Electrostatic Funnel in the GABA-Binding Pathway
Source: PLoS Comput Biol. 2016 Apr 27;12(4):e1004831. doi: 10.1371/journal.pcbi.1004831 (PMC4847780; doi:10.1371/journal.pcbi.1004831)

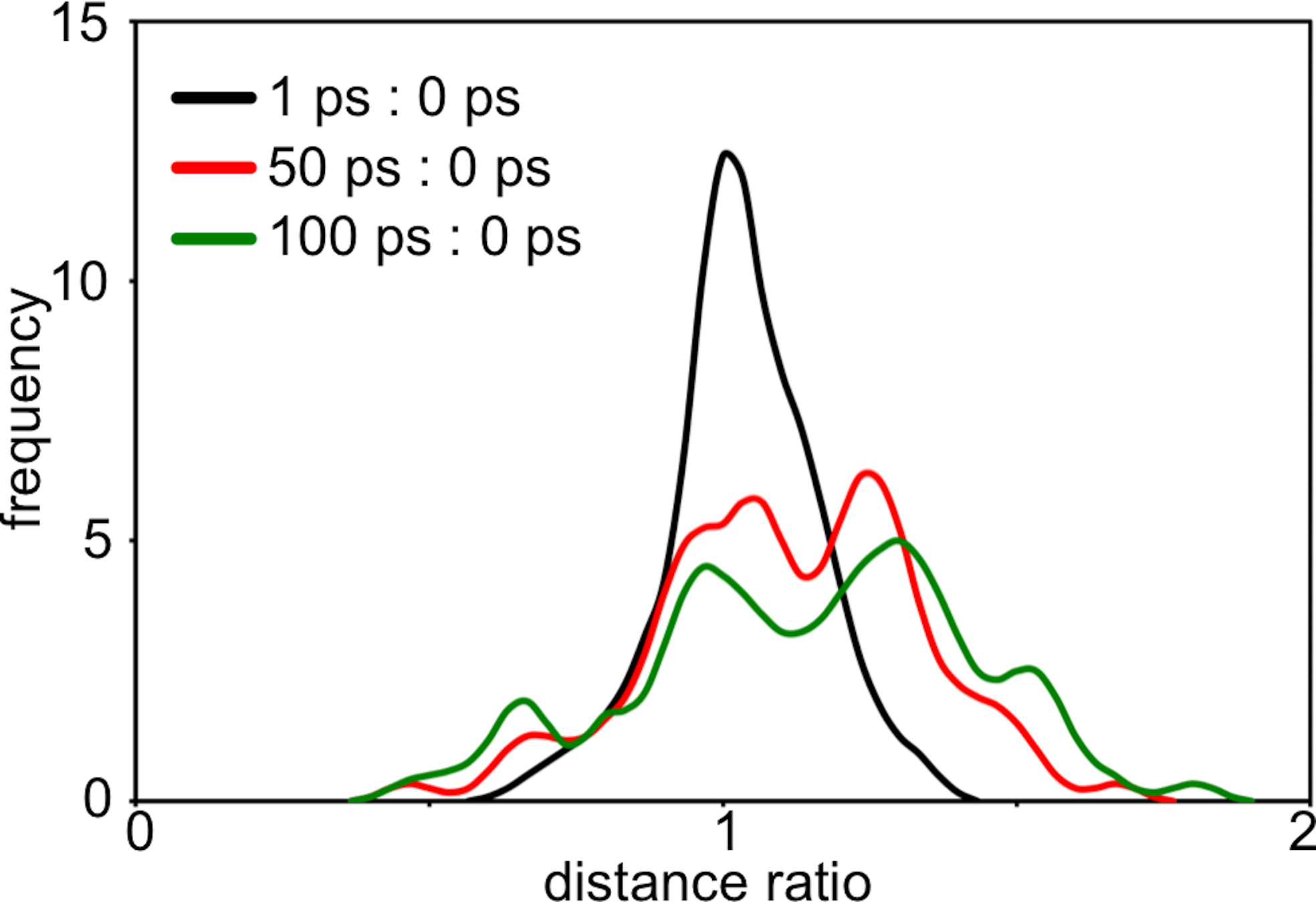

Supplement: S1 Fig — A histogram depicts the ratios of the GABA to binding site distance after 1 (black line), 50 (red line), and 100 (green line) ps compared to the initial GABA to binding site distance for all 100 simulations. (TIFF) [file pcbi.1004831.s001.tiff]

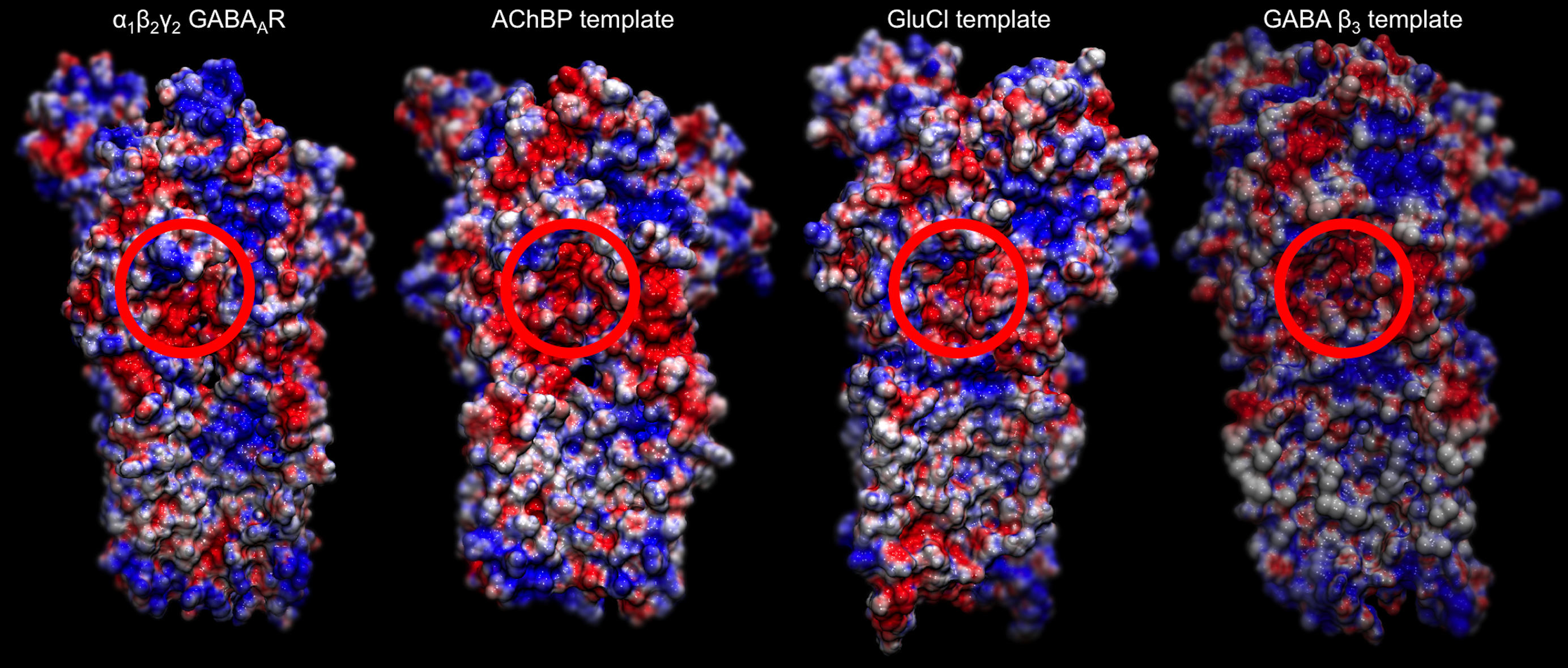

Supplement: S2 Fig — The electrostatic surfaces were calculated for various different GABAA-R homology models that were constructed using either different GABAA-R subunit sequences or different structural templates. They are (L-R): model of the alternative α1β2γ2 isoform, α6β3δ model using a complete AChBP for the LBD template, α6β3δ model using GluCl as a template, α6β3δ model using the new GABAA-R β3 homopentamer as a template. (TIFF) [file pcbi.1004831.s002.tiff]

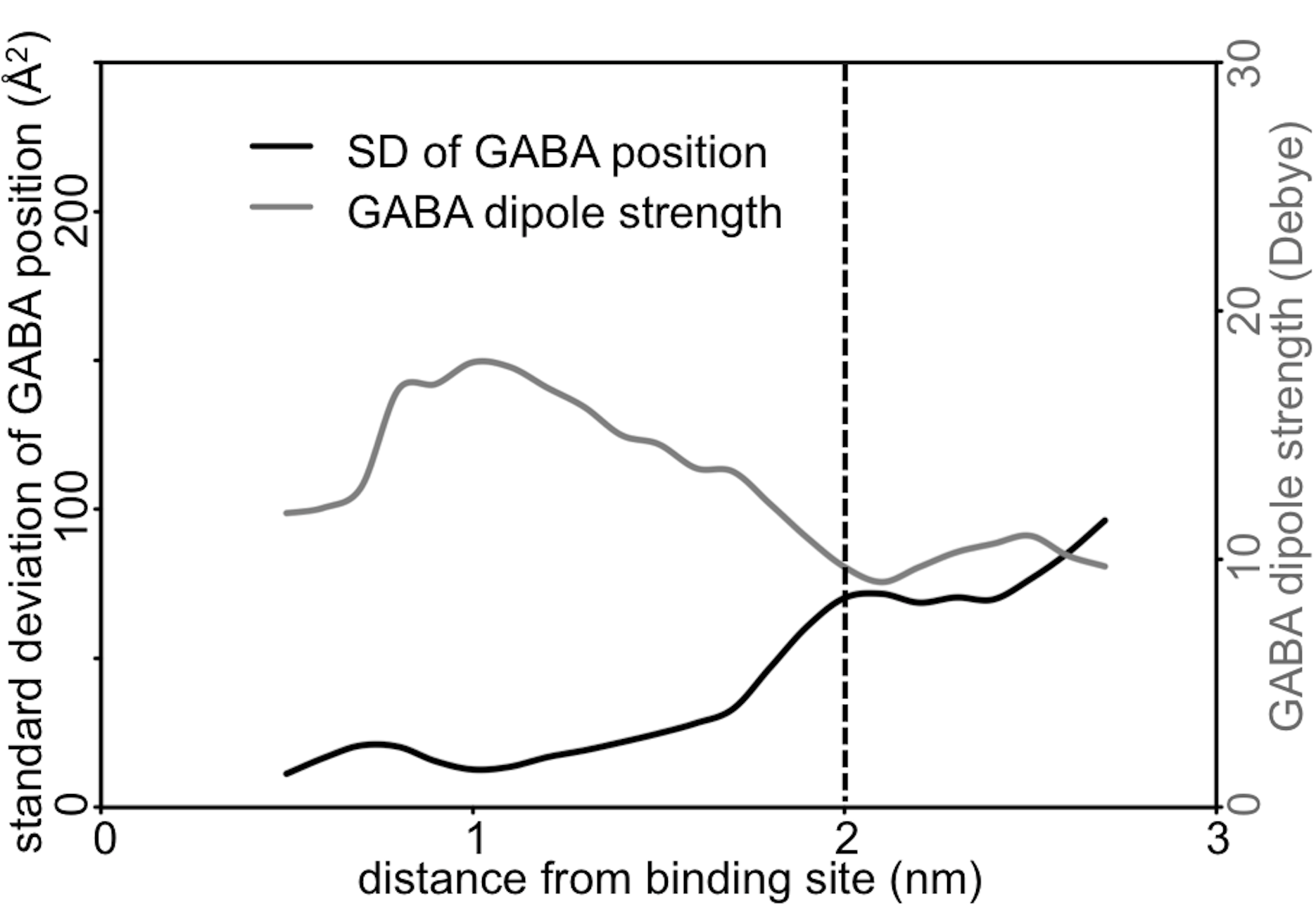

Supplement: S3 Fig — The standard deviation of the population of GABA molecules (black) and the average dipole of the GABA population (grey) both show a distinct change in behavior ~1.9–2.0 nm from the binding site COM (indicated by the dashed black line). These values were calculated using all of the binding simulations (BIND, PARTIAL, and NEARBY). (TIFF) [file pcbi.1004831.s003.tiff]

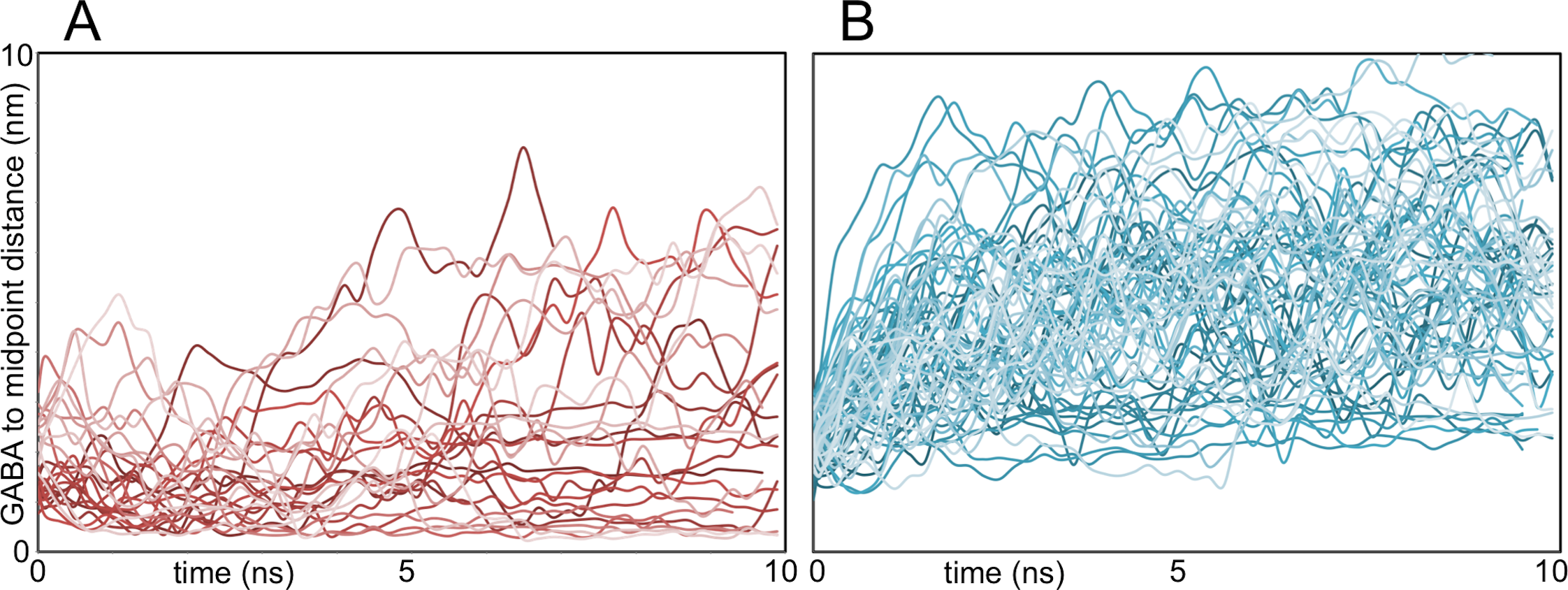

Supplement: S4 Fig — Of the 100 independent simulations, GABA accesses the midpoint area (defined as reaching a point < 1 nm away) in 28 of them (A), leaving 72 simulations where GABA does not reach the vicinity of the midpoint at all (B). (TIFF) [file pcbi.1004831.s004.tiff]

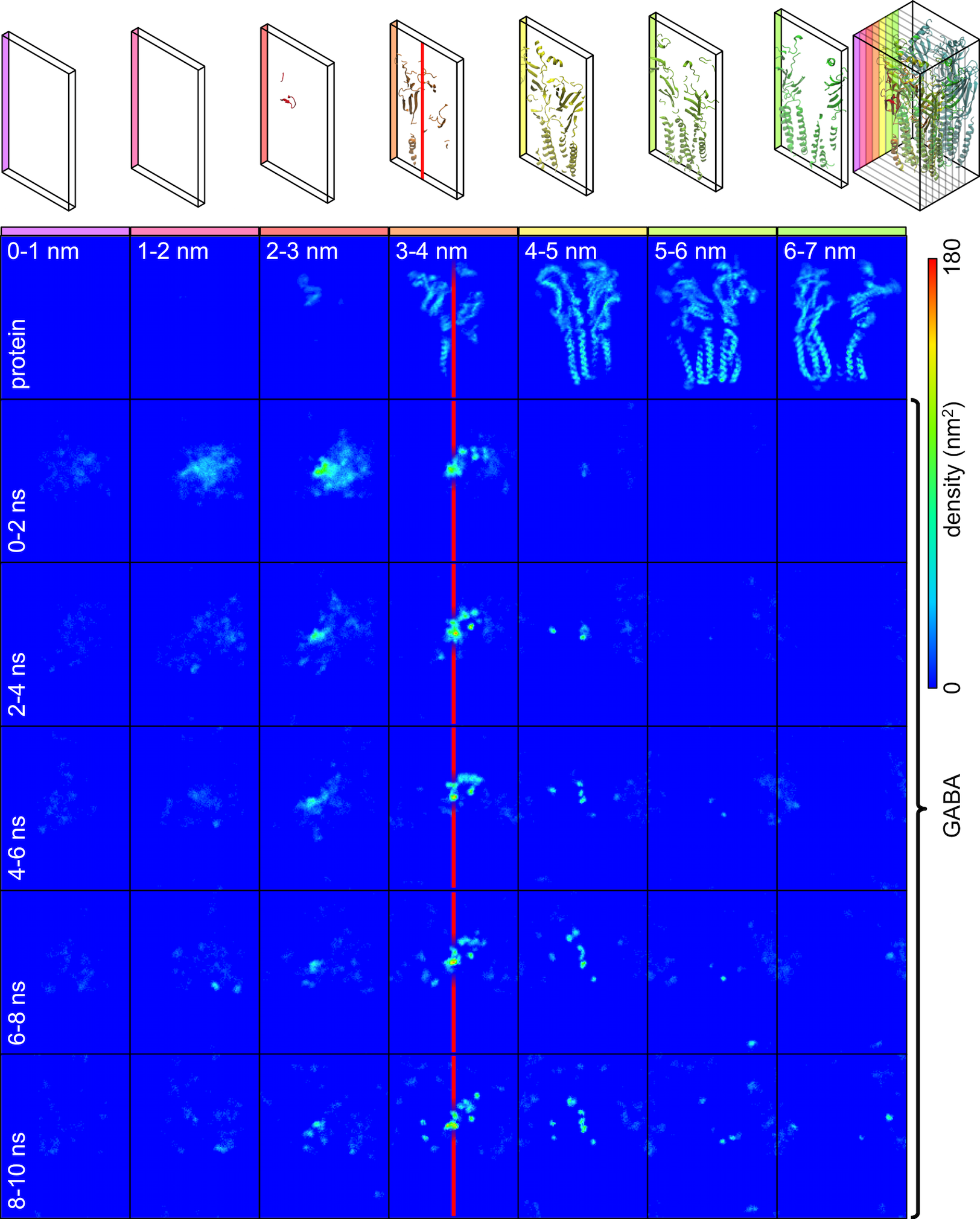

Supplement: S5 Fig — The system was divided into 1 nm thick slices starting from the front of the system and ending at the middle of the protein. The depth of the slices are indicated by a rainbow color scale. The protein density is averaged over the duration of several randomly selected simulations. The density of GABA molecules from all 100 simulations averaged over the same slice, shown as time progresses. The vertical red line through the 3–4 nm slices indicates the center of the GABA binding site. (TIFF) [file pcbi.1004831.s005.tiff]
